# Profiling spatiotemporal gene expression of the developing human spinal cord and implications for ependymoma origin

**DOI:** 10.1038/s41593-023-01312-9

**Published:** 2023-04-24

**Authors:** Xiaofei Li, Zaneta Andrusivova, Paulo Czarnewski, Christoffer Mattsson Langseth, Alma Andersson, Yang Liu, Daniel Gyllborg, Emelie Braun, Ludvig Larsson, Lijuan Hu, Zhanna Alekseenko, Hower Lee, Christophe Avenel, Helena Kopp Kallner, Elisabet Åkesson, Igor Adameyko, Mats Nilsson, Sten Linnarsson, Joakim Lundeberg, Erik Sundström

**Affiliations:** 1 Division of Neurogeriatrics, Department of Neurobiology, Care Sciences and Society, Karolinska Institutet, Stockholm, Sweden; 2 Science for Life Laboratory, Department of Gene Technology, KTH Royal Institute of Technology, Stockholm, Sweden; 3 Science for Life Laboratory, Department of Biochemistry and Biophysics, National Bioinformatics Infrastructure Sweden, Stockholm University, Stockholm, Sweden; 4 Science for Life Laboratory, Department of Biochemistry and Biophysics, Stockholm University, Stockholm, Sweden; 5 China National Clinical Research Center for Neurological Diseases, Beijing Tiantan Hospital, Capital Medical University, Beijing, China; 6 Division of Molecular Neurobiology, Department of Medical Biochemistry and Biophysics, Karolinska Institutet, Stockholm, Sweden; 7 Department of Cell and Molecular Biology, Karolinska Institutet, Stockholm, Sweden; 8 Department of Information Technology, Uppsala University, Uppsala, Sweden; 9 BioImage Informatics Facility, Science for Life Laboratory, SciLifeLab, Sweden; 10 Department of Clinical Sciences, Danderyd Hospital, Karolinska Institutet, Stockholm, Sweden; 11 Department of Obstetrics and Gynecology, Danderyd Hospital, Danderyd, Sweden; 12 R&D Unit, Stockholms Sjukhem, Stockholm, Sweden; 13 Department of Physiology and Pharmacology, Karolinska Institutet, Stockholm, Sweden; 14 Department of Neuroimmunology, Center for Brain Research, Medical University of Vienna, Vienna, Austria; 15 Present Address: Department of Artificial Intelligence and Machine Learning, Research and Early Development, Genentech. Inc., South San Francisco, CA USA

**Keywords:** Developmental biology, Cell fate and cell lineage, Gene expression, Spinal cord

## Abstract

The spatiotemporal regulation of cell fate specification in the human developing spinal cord remains largely unknown. In this study, by performing integrated analysis of single-cell and spatial multi-omics data, we used 16 prenatal human samples to create a comprehensive developmental cell atlas of the spinal cord during post-conceptional weeks 5–12. This revealed how the cell fate commitment of neural progenitor cells and their spatial positioning are spatiotemporally regulated by specific gene sets. We identified unique events in human spinal cord development relative to rodents, including earlier quiescence of active neural stem cells, differential regulation of cell differentiation and distinct spatiotemporal genetic regulation of cell fate choices. In addition, by integrating our atlas with pediatric ependymomas data, we identified specific molecular signatures and lineage-specific genes of cancer stem cells during progression. Thus, we delineate spatiotemporal genetic regulation of human spinal cord development and leverage these data to gain disease insight.

## Abstract (web summary)

The authors created a comprehensive developmental cell atlas for spatiotemporal gene expression of the human spinal cord, revealed species-specific regulation during development and used the atlas to infer novel markers for pediatric ependymomas.

## Main

The spinal cord comprises the caudal region of the central nervous system (CNS) and is responsible for conveying and processing motor and sensory information between the brain and the periphery. During spinal cord development, gradients of dorsal and ventral morphogens^1^ regulate the cell fate commitment of neural stem and progenitor cells (NPCs) in the ventricular zone surrounding the nascent central canal. Various transcription factors (TFs) along the dorsal–ventral (DV) axis are then activated, resulting in spatially segregated progenitor domains. In rodents, domain-specific NPCs first generate neurons and then glia, and these differentiated neural cells migrate to their final locations in the spinal cord and form distinct circuits^1^.

It is, however, not known to what extent this knowledge can be extended to humans. It is generally thought that, during the first trimester of pregnancy, most of the human NPCs (hNPCs) are highly proliferative in preparation for neurogenesis and gliogenesis. Recent studies, however, showed that hNPCs derived from early development exhibit either robust glial differentiation^2^ or little differentiation^3^, suggesting that more information about the genetic regulation of cell fate commitment in hNPCs is necessary. Furthermore, this information may provide insight into pediatric tumorigenesis and neurodevelopmental disease.

Single-cell RNA sequencing (scRNA-seq) and spatial transcriptomics (ST) have provided high-throughput and spatially resolved analysis of gene expression during human prenatal development^4^. Furthermore, a high-throughput and multiplex in situ hybridization method, called hybridization-based in situ sequencing (HybISS), has recently been developed for single RNA molecule localization of large gene panels with single-cell resolution within human tissue for data validation^4,5^. Combining these methods can help overcome the limitations of individual techniques, facilitate unbiased cell type annotation and allow high-resolution spatiotemporal mapping of the developing human spinal cord. Two recent studies used scRNA-seq on human developing spinal cord and revealed the appearance of different neural cell types^6,7^. However, the genetic regulation of the commitment of homogenous hNPCs to heterogenous neuronal and glial fates in vivo is still unclear. Furthermore, although neural patterning during human development is well described^7,8^, how neural patterning directs regional neuronal and glial differentiation is still not well studied in human.

In this study, we analyzed 16 human embryonic and fetal spinal cord samples, with ages spanning across the first trimester, from post-conceptional week (W) 5–12, using scRNA-seq, ST and HybISS, and we integrated these datasets with previously reported mouse and human spinal cord datasets. Here we provide a comprehensive developmental cell atlas of the human spinal cord, reveal spatiotemporal gene expression and regulation of cell fate commitment, highlight the major differences of cellular and molecular events in human and rodent spinal cord development and report the discovery of novel molecular targets and genetic regulation of pediatric spinal cancer stem cells (CSCs).

## Results

### Comprehensive atlas of the human developing spinal cord

**Fig. 1:**
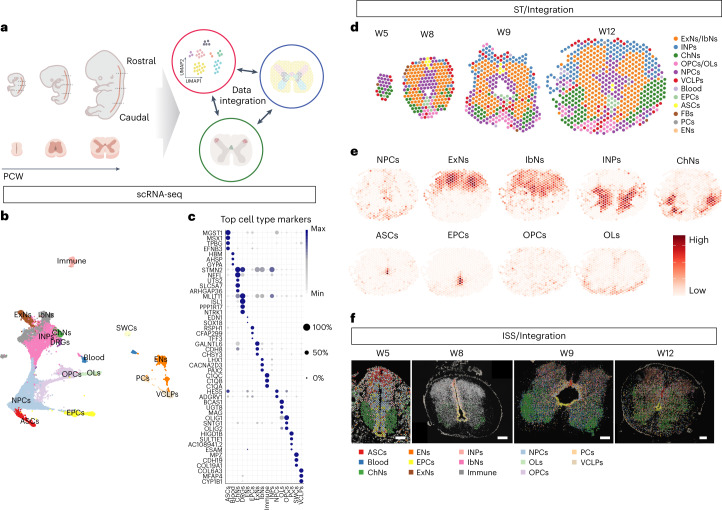
Comprehensive atlas of the developing human spinal cord. **a**, Schematic overview of the workflow. **b**, UMAP of scRNA-seq datasets revealing major cell populations. **c**, Dot plot illustrating top marker genes for major cell populations. **d**, Spatial mapping of major cell types from ST analysis in representative human spinal cord sections. **e**, Representative stereoscope plots of one W12 section. **f**, Representative images and cell typing results from HybISS. Scale bar, 200 μm. Two independent experiments were performed. PCW, post-conception week.

**Extended Data Fig. 1:**
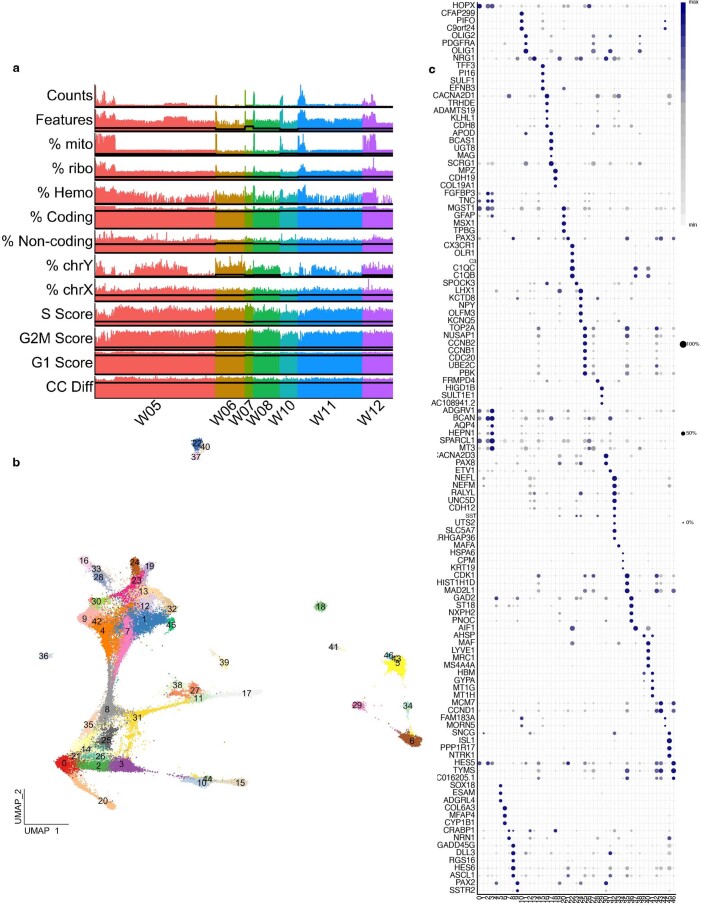
scRNA-seq reveals cell heterogeneity of the developing human spinal cord. **a)** Quality control and filtering strategies for scRNA-seq dataseq. Thick lines indicate filter thresholds. **b)** UMAPs identifying 47 clusters (cluster 0-46). **c)** Dot plot illustrating top marker genes of each cluster. In relation to Fig. 1.

To investigate the molecular features of the developing human spinal cord, we acquired 16 human prenatal spinal cords at W5–12 (Supplementary Table 1), covering the first trimester of pregnancy when cell fate specifications in the CNS occur^9,10^. We performed scRNA-seq, ST and HybISS to create a developmental cell atlas of the human spinal cord with detailed spatiotemporal gene expression and validation (Fig. 1a and Supplementary Table 1). A total of 159,350 high-quality cells across 31 scRNA-seq libraries were analyzed, revealing 47 cell clusters (Extended Data Fig. 1a,b) (16 major cell populations) (Fig. 1b). All major spinal cord neural cell types were represented, including NPCs, intermediate neuronal progenitors (INPs), excitatory neurons (ExNs), inhibitory neurons (IbNs), cholinergic neurons (ChNs), astrocytes (ASCs), ependymal cells (EPCs), oligodendrocyte precursor cells (OPCs) and oligodendrocytes (OLs) (Fig. 1b), which this study mainly focused on. Other cell types, such as Schwann cells (SWCs), pericytes (PCs), endothelial cells (ENs), vascular capillary endothelial cells (VCLPs) and immune cells (Immune) (for example, microglia), were also derived during this developmental stage (Fig. 1b). Top marker genes of each cell type and cluster are summarized (Fig. 1c and Extended Data Fig. 1c).

**Extended Data Fig. 2:**
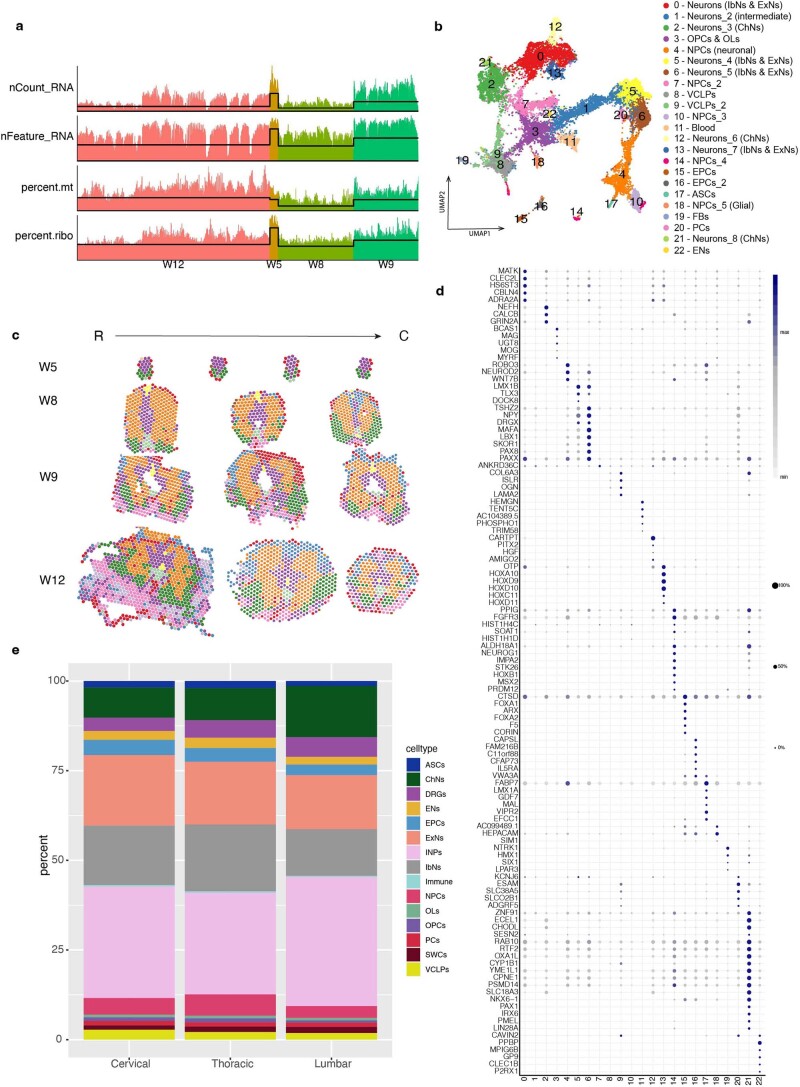
Temporal and spatial gene expression in the developing human spinal cord by ST. **a)** Quality control results of ST. Lines indicate filtering thresholds. **b)** UMAP illustrating 23 clusters from 76 ST sections at W5, W8, W9 and W12. **c)** Representative sections of ST spatial maps of all clusters along rostral-caudal axis. R = rostral, C = caudal. **d)** Dot plot illustrating the top marker genes for all clusters in the ST analysis. **e)** Bar graph illustrating cell type proportions across sections along rostral-caudal axis. In relation to Fig. 1.

**Extended Data Fig. 3:**
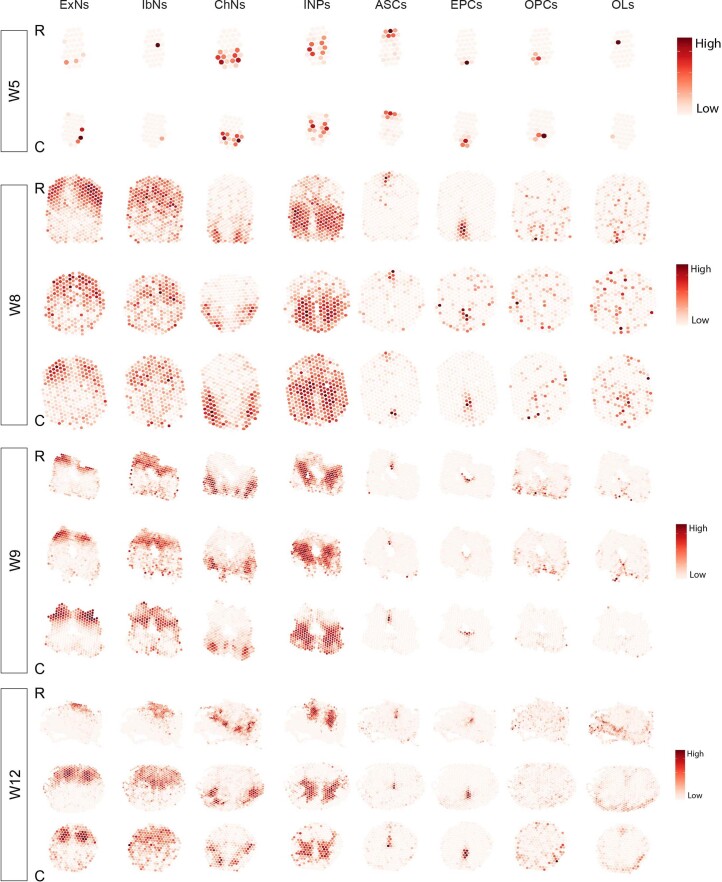
Stereoscope revealing integrated results of scRNA-seq and ST. Representative stereoscope images revealing cell type positions and their probability along the rostral-caudal axis at W5, 8, 9 and 12. R = rostral, C = caudal. Intermediate neuronal progenitors (INPs), excitatory neurons (ExNs), inhibitory neurons (IbNs), cholinergic neurons (ChNs), astrocytes (ASCs), ependymal cells (EPCs), oligodendrocyte precursor cells (OPCs), oligodendrocytes (OLs). In relation to Fig. 1.

To define the spatial gene expression and cell type localization, we analyzed sections of the prenatal spinal cords along the rostral–caudal (RC) axis of representative ages (W5, W8, W9 and W12). Seventy-six sections from ST resulted in 23 clusters (12 major cell types) along the RC and DV axis (Extended Data Fig. 2a–b,d) (Fig. 1d and Extended Data Fig. 2c). At W5, the cross-sectioned human spinal cord was dominated by NPCs in the ventricular zone. From W8 and onwards, neurons as well as all glial cell types were found (Fig. 1d and Extended Data Fig. 2c). In addition, fewer cell types could be identified in the caudal regions (for example, cluster 0 neurons at W8) compared to the rostral regions, suggesting an earlier development in rostral regions (Extended Data Fig. 2c,e). However, no obvious differences in gene expression from different regions along the RC axis were found, possibly related to protein changes. To understand the probability of cell types in different of the human spinal cord, we integrated scRNA-seq and ST data by using stereoscope, a method for guided decomposition of ST data by using scRNA-seq data as reference^11^ to delineate the spatial distribution of cell types defined in the scRNA-seq. (Fig. 1e and Extended Data Fig. 3). Notably, stereoscope data indicate the relative probability of each cell type in certain spot rather than an absolute value of cell number quantification. To provide single-cell spatial mapping resolution and validation, we performed HybISS^5^ in adjacent tissue sections using 50 selected genes (Supplementary Table 3 and Supplementary Fig. 1) for major cell type characterization and 224 genes for subtype or cell state characterization (Supplementary Table 4 and Supplementary Fig. 2). The HybISS data were integrated with scRNA-seq data by probabilistic cell typing (pciSeq)^12^ and confirmed the findings revealed by ST (Fig. 1f). Notably, either ST or HybISS was also analyzed independently from scRNA-seq data as validation (more detail below).

## Diverse neural cells in the human developing spinal cord

**Fig. 2:**
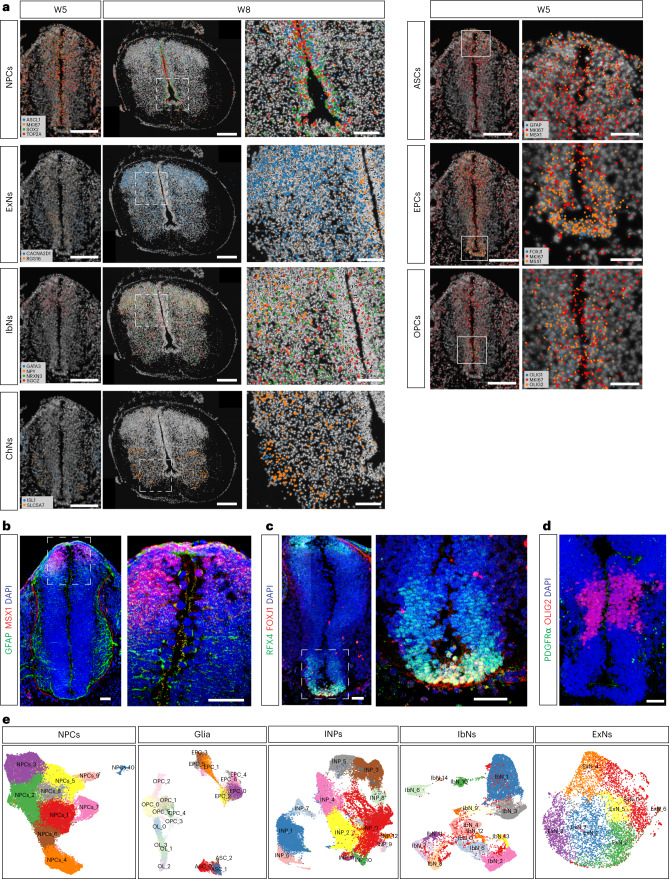
Heterogenous neural cells in the human developing spinal cord. **a**, Representative images showing validation of newborn neurons and glial cells in the developing human spinal cord by HybISS. Scale bar, 200 μm. **b**, Representative confocal images showing immunostaining of newborn astrocytes (**b**) and ependymal cells (**c**) at W5, whereas OPCs are not born at W5 yet (**d**). Two independent experiments for each panel (**a**–**d**) were performed. Scale bars, 200 μm and 50 μm for low and high magnification, respectively. Rectangles indicate enlarged areas. **e**, UMAP illustrating the heterogenous cell types or cell states of different neural cell populations.

**Extended Data Fig. 4:**
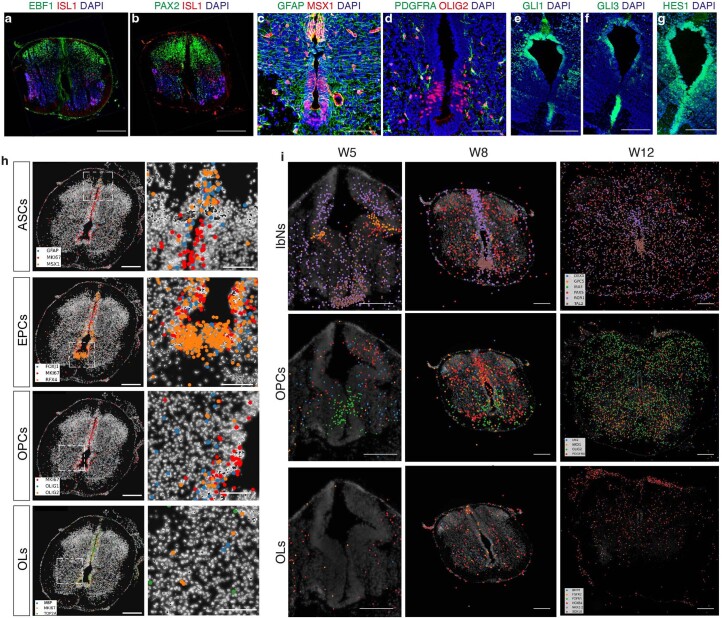
Validation of cell populations, cell fate commitment and neural patterning at early developmental stage. **a–g**) Representative IHC images illustrating localization of ExNs, IbNs and ChNs (a-b), ASCs (c) and OPCs (d) in W8 human spinal cord as well as SHH and Notch related proteins at W5 human spinal cord sections (e-g). **h)** Representative HybISS images illustrating early glial cells at W8. Rectangles indicating enlarged areas. **i)** Representative HybISS images illustrating localization of subpopulations of IbNs, OPCs and OLs during human spinal cord development. Scale bars: 100 μm. In relation to Figs. 2 and 4. Two independent experiments for each panel were performed.

To validate the major cell populations identified by scRNA-seq and ST (Fig. 1b,d,e), our HybISS data confirmed ST results (Fig. 1d,e): NPCs (*ASCL1*^+^*SOX2*^+^) were the major cell population at W5 and were highly proliferative (*MKI67*^+^*TOP2A*^+^) but were restricted to the ventricular zone from W8 (Fig. 2a). Neurons, including ExNs (*CACN2D1*^+^), IbNs (*SCGZ*^+^ or *NRXN3*^+^) and ChNs (*ISL1*^+^ and/or *SLC5A7*^+^), appeared as early as W5 and were widely distributed throughout the gray matter (that is, the intermediate zone) at W8 (Fig. 2a), in line with ST data (Fig. 1d). Immunohistochemistry (IHC) confirmed early neurogenesis at W8 for ExNs (EBF1^+^), IbNs (PAX2^+^) and ChNs (ISL1^+^) (Extended Data Fig. 4a,b). Although a previous study on human developing spinal cord showed that glial cells first appeared at W7–8 (ref. ^6^), we observed that all glial cell markers were expressed at W5, in which ASCs (*MSX1*^+^*GFAP*^+^) were derived from the dorsal ventricular zone; EPCs (*FOXJ1*^+^*RFX4*^+^) were derived from the ventral ventricular zone; and OPCs (*OLIG1*^+^*OLIG2*^+^) were derived from the pMN domain (Fig. 2a). All these glial cell types showed *MKI67* expression, suggesting that gliogenesis continued from W5–8 on (Fig. 2a and Extended Data Fig. 4h). In addition, we observed IHC signals at W5 for ASCs (MSX1^+^GFAP^+^) and EPCs (RFX4^+^FOXJ1^+^) in the dorsal and ventral area of the spinal cord, respectively, in agreement with HybISS results (Fig. 2b,c). However, we did not observe OPC markers at the protein level (PDGFRa^+^OLIG2^+^) until W8 (Fig. 2d and Extended Data Fig. 4d). Our data suggest that NPCs were committed to glial fate as early as W5 in the developing human spinal cord.

**Extended Data Fig. 5:**
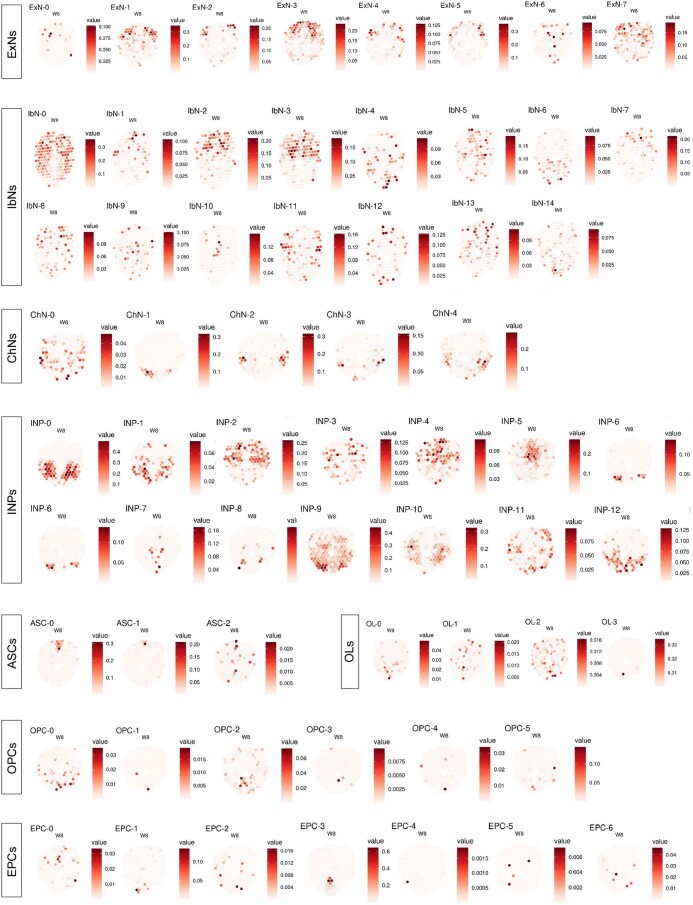
Locolization of heterogenous cell types or cell states of neural cells in the human developing spinal cord. Representative images from stereoscope analysis illustrating the probability of spatial distribution of each cell subpopulation or cell state of the major neural cells at W8. In relation to Fig. 2.

To further characterize the heterogenous cell types and cell states, we analyzed each major neural cell type to reveal their diversity (Fig. 2e). These subpopulations or cell states could be distinguished with single or combinatorial markers (Supplementary Fig. 2). We then used stereoscope to determine their spatial distribution (Extended Data Fig. 5). Some neurons exhibited specific spatial distributions, such as IbNs_2 in the dorsal parts and IbNs_6 in the ventral parts. Similarly, the early born glial cells showed specific spatial distributions, with EPCs_0 in the dorsal ventricular zone and EPCs_3 in the ventral ventricular zone (Extended Data Fig. 5). We also validated the regional distribution of subclusters by HybISS, such as IbNs_6 (*TAL2*^+^) in the ventral spinal cord (Extended Data Figs. 5 and 4i) and IbNs_13 in the dorsal-central spinal cord with *GPC5*, *DTX1* and *ROR1* expression (Extended Data Figs. 5 and 4i). For glial cells, most OPCs were derived from the ventral spinal cord (Fig. 2a and Extended Data Fig. 5) and were *PDGFRA*^+^*OLIG2*^+^, with exceptions of OPCs_2 (PDGFRA^−^*OLIG2*^+^*NKD1*^+^) and OPCs_3 (*EN2*^+^) (Extended Data Fig. 4i and Supplementary Fig. 2). Notably, many clusters of neuronal and glial cell types did not display regionally specific distributions, suggesting that these subclusters represent transient cell states. Indeed, by performing Gene Ontology (GO) analysis on the differentially expressed genes (DEGs) of different neural clusters, the most common results were associated with ‘neurodevelopment’, ‘neurogenesis’ and ‘gliogenesis’. Therefore, we focused on how neurogenesis and gliogenesis are regulated by spatiotemporal gene expression during NPC self-renewal, fate commitment and differentiation in the developing human spinal cord.

## NPC commitment to neural fates at early stages

**Extended Data Fig. 6:**
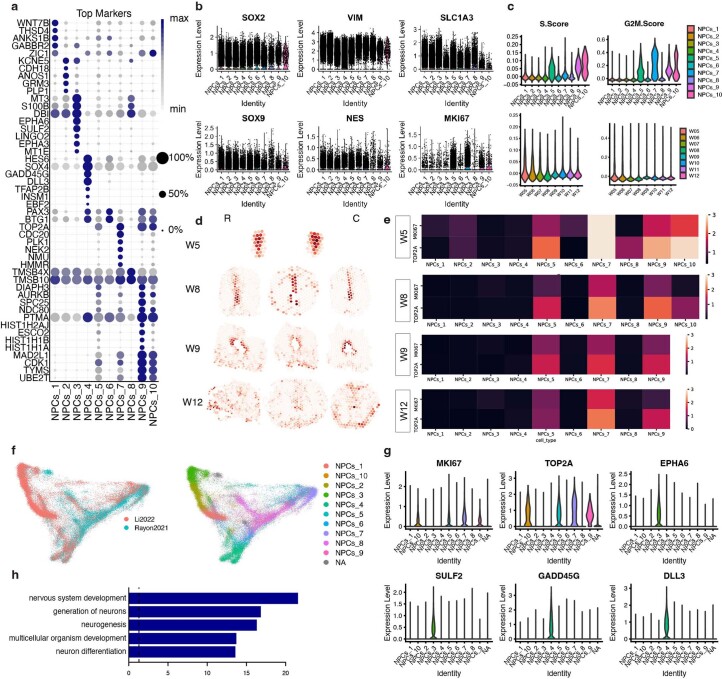
Heterogenous NPCs with different activeness in the developing human spinal cord. **a)** Dot plot illustrating the top markers for the NPC clusters. **b-c**) Violin plots illustrating stem cell markers (b) and cell cycle scores (c) of human NPCs across clusters and ages. **d)** Stereoscope illustrating the probability of spatial distribution of different NPCs in the developing human spinal cord sections. **e)** HybISS illustrating the locations of the proliferative NPC clusters from W5-12. Scale bar 100μm. **f)** UMAP illustrating integrated datasets and subtypes of NPCs. **g)** Violin plots illustrating consistent results for gene expression of proliferation markers and subtype specific markers in the integrated dataset. **h)** Top GO terms of early non-proliferative NPCs compared to proliferative NPCs. In relation to Figs. 2 and 3.

In the analysis of cell fate commitment, we first focused on the NPC population with 10 different clusters in the scRNA-seq dataset (Fig. 2e and Extended Data Fig. 6a), with overall expression of neural stem cell markers, indicating their stem cell properties (Extended Data Fig. 6b). In contrast to the common view that most embryonic/fetal NPCs proliferate extensively^1^, we found that more than half of the clusters expressed low levels of active cell cycle genes (S or G-to-M phase) (Extended Data Fig. 6c). Spatially, hNPCs were mostly located around the ventricular zone at W5 and then distributed in intermediate zones from W9 to W12, probably due to migration (Extended Data Fig. 6d). Interestingly, HybISS data showed that the expression of proliferation markers (*MKI67* and *TOP2A*) substantially decreased in hNPCs, and hNPCs_10 even disappeared from W9 (Extended Data Fig. 6e). In agreement, IHC showed that many SOX9^+^ hNPCs did not express Ki-67 in the W5 human spinal cord, suggesting that a large proportion of hNPCs enter quiescence in early development (Fig. 5a).

**Fig. 3:**
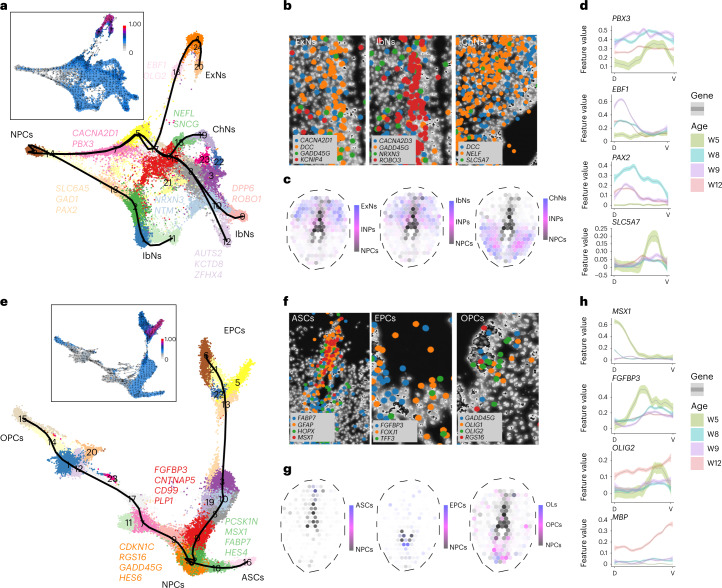
Spatiotemporal regulation of human neurogenesis and gliogenesis. **a**, UMAP displaying branches from NPCs to different neuronal clusters, confirmed by RNA velocity (left upper panel). Lighter colors indicate undifferentiated states; darker colors indicate differentiating states. **b**, HybISS revealing the co-location of NPCs, neuronal markers and lineage-related genes revealed by trajectory analysis. **c**, Integrated trajectory and ST data revealing neuronal spatial differentiation. **d**, Spatial quantification of neuronal lineage-associated gene expression along the DV axis across ages. **e**, UMAP indicating branches from NPCs to different glia, confirmed by RNA velocity (upper panel). **f**, HybISS revealing the co-location of NPCs, glial markers and lineage-related genes. **g**, Integrated trajectory and ST data revealing glial spatial differentiation. **h**, Spatial quantification of the expression of glial lineage-associated genes along the DV axis across ages.

**Extended Data Fig. 7:**
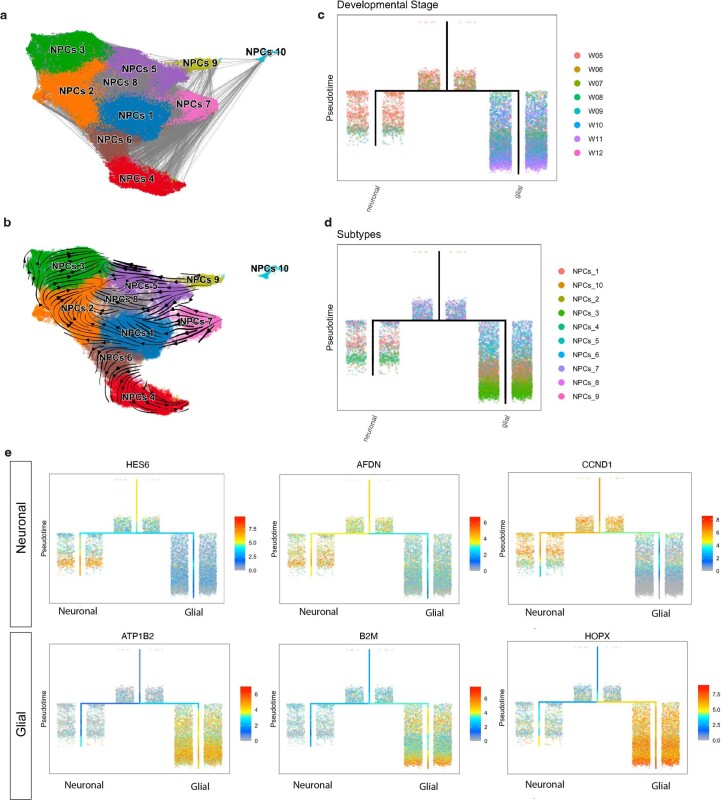
Cell fate commitment of hNPCs during human spinal cord development. **a)** UMAP showing strong connectivity of different NPC clusters during development. **b)** scVelo analysis revealing the predicted differentiation trajectory from proliferative NPCs to neuronal and glial fate committed NPCs. **c-d**) Hierarchical tree from URD analysis displaying NPC trajectory during development. **e)** Hierarchical trees illustrating examples of top genes associated with neuronal and glial lineage during cell fate commitment of hNPCs. In relation to Fig. 3.

To analyze the starting point of differentiation, we used two different methods for trajectory analysis—scVelo^13^ (Extended Data Fig. 7a,b) and URD^14^ (Extended Data Fig. 7c–e)—on the NPC populations. All NPC populations were highly connected with each other (Extended Data Fig. 7a), and the proliferative hNPCs (NPCs_5, 7, 9 and 10) changed their fates toward low-proliferating NPC clusters (NPCs_3 and 4) and further into neurons and glia (Extended Data Fig. 7b; more details in Fig. 3). Different genes including TFs were specifically associated with either neuronal or glial lineages (Extended Data Fig. 7e), suggesting that most NPCs were genetically regulated for fate commitment into either neurons or glia at W5 (Extended Data Fig. 7c–e). We confirmed these observations by integrating our scRNA-seq dataset with data from W4–7 spinal cord^7^ (Extended Data Fig. 6f) and showed consistent results (Extended Data Fig. 6g), in line with our own NPC data (Extended Data Fig. 6a). By selecting NPCs from the earliest stages (W5 and Carnegie stage (CS) 12), we compared the DEGs of non-proliferative versus proliferative NPCs and found that neuronal differentiation and neurogenesis were the top GO terms (Extended Data Fig. 6h), suggesting that non-proliferative hNPCs were involved in differentiation, in line with our trajectory analysis of NPCs.

## Spatiotemporal gene expression regulates neurodevelopment

**Extended Data Fig. 8:**
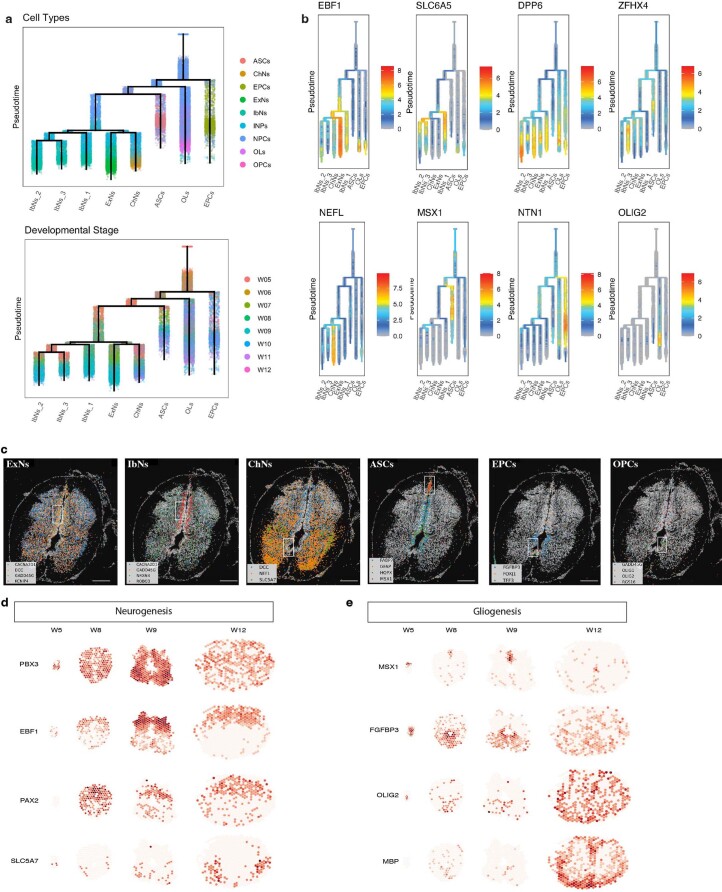
Spatiotemporal regulation of human neurogenesis and gliogenesis. **a-b**) Hierarchical trees from URD analysis displaying neurogenesis and gliogenesis (a) as well as top lineage-associated genes (b) during human spinal cord development. **c)** Representative HybISS images illustrating the co-locolization of NPC markers, committed cell fate markers and lineage-associated genes. Scale bar 100μm. Two independent experiments were performed. **e)** ST plots illustrating the spatial expression of top lineage genes revealed by scRNA-seq. In relation to Fig. 3.

To characterize NPC differentiation, we selected related NPC and neuron clusters from the scRNA-seq data for three trajectory analysis methods: Slingshot, RNA velocity and URD^14–16^ (Fig. 3a,e and Extended Data Fig. 8a,b). Slingshot revealed NPC differentiation into multiple neuronal lineages (Fig. 3a,b and Supplementary Fig. 3a,b), with specific gene expression associated with each branch (Fig. 3a and Supplementary Fig. 3c,d). HybISS further validated the trajectory results by showing different newborn neurons co-expressed NPC marker genes (*DCC* and *GADD45G*), neuronal lineage-associated genes (*CACNA2D1* in ExNs, *NRXN3* in IbNs and *NEFL* in ChNs) and neuronal markers (*KCNIP4* in ExNs, *ROBO3* in IbNs and *SLC5A7* in ChNs) (enlarged areas shown in Fig. 3b; overview in Extended Data Fig. 8c).

To further delineate neuronal differentiation spatially, we integrated our scRNA-seq trajectory and ST data and showed that hNPCs differentiated into INPs first and then into different functional neurons (Fig. 3c). Furthermore, to validate these spatial trajectory calculations, we developed a method and implemented it as an R package that allowed us to spatially quantify gene expression along the DV axis in the ST dataset. We found that the most significant temporal lineage-associated genes revealed by scRNA-seq, such as *EBF1* (for ExNs), *PAX2* (for IbNs) and *SLC5A7* (for ChNs), exhibited a biased DV expression in ST analysis, which correlated with the localization of differentiated neuron types (Fig. 3d and Extended Data Fig. 8d). *PBX3* was associated with all three neuronal lineages and, thus, did not exhibit a specific DV pattern from W8 (Fig. 3d). The results were additionally confirmed by the gene expression pattern in HybISS (Extended Data Fig. 8c) and reveal the spatiotemporal gene expression associated with neurogenesis.

For gliogenesis, we performed a similar analysis and showed that all three glial lineages originated from one common NPC subtype (Fig. 3e and Extended Data Fig. 8a,b), with specific branch-associated genes (for example, *CNTNAP5* for EPCs, *MSX1* for ASCs and *HES6* for OPCs) (Fig. 3e, Extended Data Fig. 8b and Supplementary Fig. 3e,f). Spatially, these lineage-associated genes were expressed in the same area as the newborn glial cells (Fig. 3f and Extended Data Fig. 8c). Integrated trajectory and ST data showed that hNPCs differentiated into glial cells in specific spatial patterns—ASCs in the dorsal spinal cord, EPCs in the central spinal cord and OPCs and OLs in the ventral spinal cord (Fig. 3g). We then quantified the spatial expression of these top lineage-associated genes along the DV axis and found that *MSX1*, *FGFBP3* and *OLIG2* were differently expressed (Fig. 3h and Extended Data Fig. 8e). The spatial expression pattern of *MSX1* and *FGFBP3* suggested that the patterning of newborn ASCs and EPCs mainly took place before W8. In contrast, *OLIG2* continued to show high ventral expression, and the mature OL-associated gene *MBP* exhibited strong ventral expression at W12, which correlated with the appearance of newborn mature OLs in the ventral spinal cord at W12 (Extended Data Fig. 3). These data were validated by both ST (Extended Data Fig 8e) and HybISS (Extended Data Fig. 8c).

**Extended Data Fig. 9:**
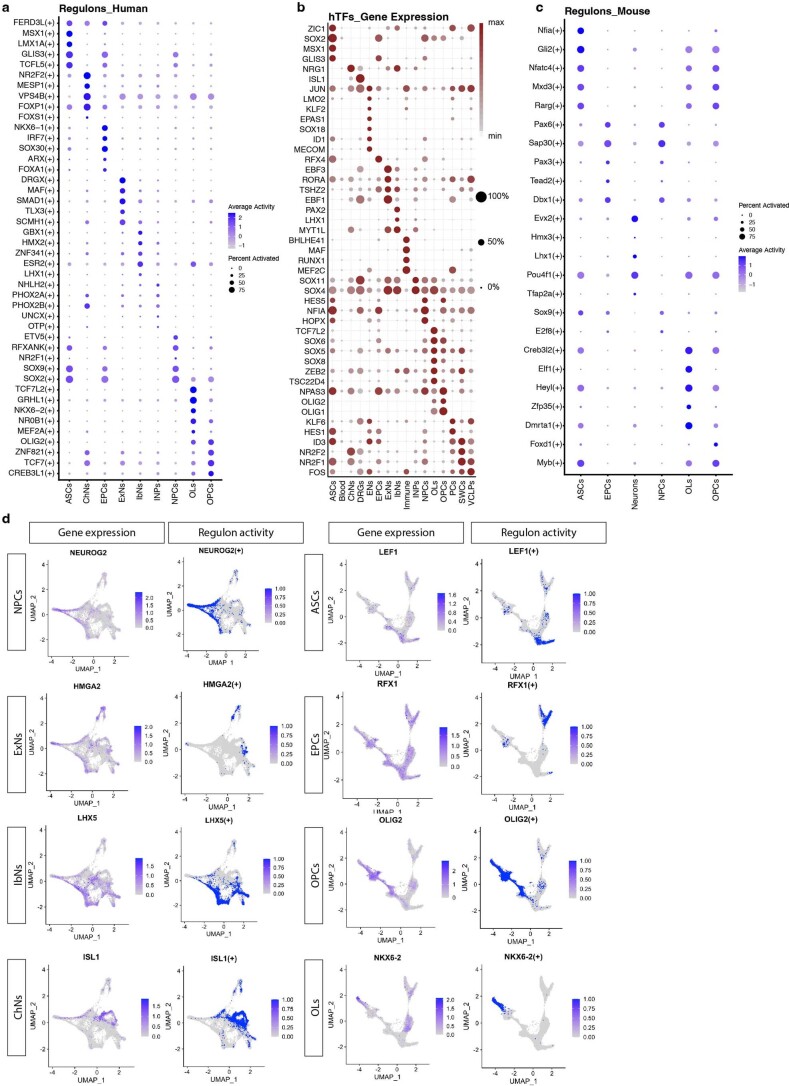
Lineage associated regulons have species difference during neurodevelopment. **a-b**) Dot plots illustrating top regulons (a) and their gene expression (b) in human major cell types during development. **c)** Dot plot illustrating top regulons during mouse spinal cord development. d) Dot plots illustrating top regulons and their gene expression during human neurogenesis and gliogenesis. In relation to Fig. 5.

To further analyze the active TFs that regulate cell fate commitment, we performed regulon analysis by SCENIC^17^ in the scRNA-seq dataset. The analysis showed the top regulons for human spinal cord development as well as the gene expression of the top TFs (Extended Data Fig. 9a,b,d). Most of the regulons for glial cells had been active since W5 (Supplementary Fig. 5a), indicating that both neuronal and glial fate commitment of NPCs occurred at this early stage, in line with HybISS and IHC data above for early glial cells at W5 (Fig. 2a–d and Extended Data Fig. 4h). Altogether, our analysis showed that the fate commitment of hNPCs is spatiotemporally regulated by specific gene sets in the developing human spinal cord.

## Genetic regulatory networks of spinal cord development

**Fig. 4:**
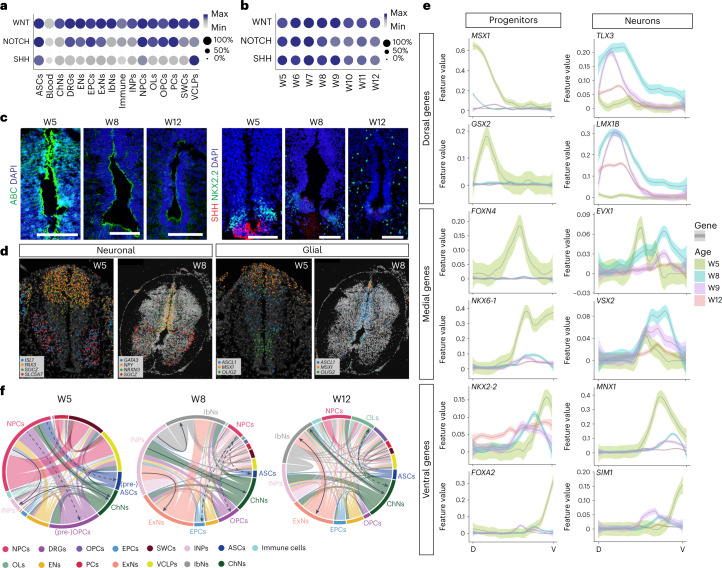
The regulatory networks of human spinal cord development. **a**,**b**, Dot plots illustrating the expression of the three major signaling pathways involved in spinal cord development in different cell types (**a**) and their decreasing expression during development (**b**). Max = highest expression of the given gene or module; Min = 0. **c**, Representative confocal images of immunostained ABC and SHH during human spinal cord development from W5 to W12. **d**, HybISS showing neuronal and glial progenitor patterning. Two independent experiments for each panel (**c** and **d**) were performed. **e**, Examples of spatial quantification of neural patterning genes along the DV axis across ages. Scale bar, 100 μm. **f**, Circos plots displaying co-localization and major connections of different cell types during development. Solid lines, neurons; dashed lines, glia.

**Extended Data Fig. 10:**
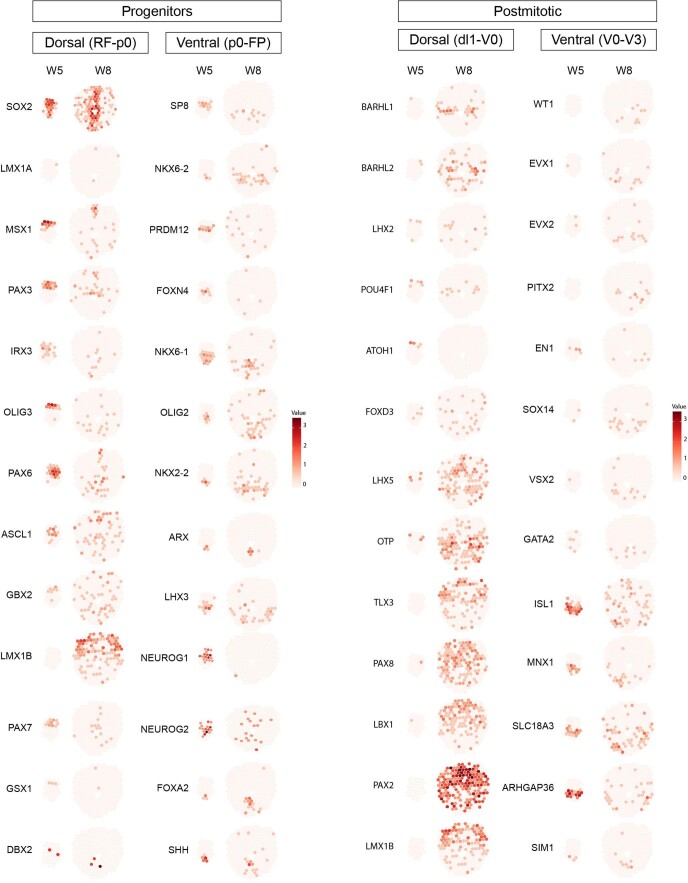
Neural patterning gene expression in the early developmental human spinal cord. Neural patterning genes related to progenitor and postmitotic neurons are plotted in W5 and W8 representative human spinal cord sections along the dorsal-ventral axis. Most of the neural patterning genes enriched in the progenitors appear at W5 but most of them disappear at W8 (Left panel). In contrast, neural patterning genes expressed in neurons are mostly absent at W5 but exhibit a dorsal-ventral patterns at W8. In relation to Fig. 4.

To better understand the regulatory control (for example, expression of TFs, morphogens, signaling pathways and cell–cell interactions) along the DV axis^1,18^, we first surveyed the most well-known signaling pathways for neural patterning and found that the gene modules of WNT, NOTCH and SHH signaling were expressed by most cell types (Fig. 4a) but overall decreased over time (Fig. 4b and Supplementary Fig. 4). IHC confirmed that active-β-catenin (ABC) and SHH pathway molecules (SHH, GLI1 and GLI3) were expressed in the roof plate and floor plate, respectively, at W5 (Fig. 4c and Extended Data Fig. 4e,f), but the expression decreased markedly after W8 (Fig. 4c). However, the NOTCH target HES1 showed overall high expression level throughout the ventricle layer, without much DV-biased expression (Extended Data Fig. 4g). Under the gradients of morphogens such as SHH and WNT, genes associated with neurogenesis and gliogenesis exhibited spatially specific expression patterns at W5 and W8, which coincided with the spatial positions of their related differentiated cell types, shown by HybISS (Fig. 4d). As variable genes used for analysis in the scRNA-seq are dominated by differentiation^7,19^, we used ST to directly measure the gene expression of neural patterning and created a detailed spatial gene expression panel that indicates the DV patterning at early stage (W5–8) (Fig. 4e and Extended Data Fig. 10). We also quantified the neural patterning gene expression and showed that their spatially unique expression was also restricted to certain developmental stages (that is, progenitor patterning genes showing DV-biased expression at W5 and neuronal patterning at W5–8) (Fig. 4e).

We next performed a co-localization analysis using the proportion estimates obtained from stereoscope to visualize the initiation of cell fate transition locally before migration starts. The ratio of NPCs decreased markedly during development (Fig. 4f). The major connections between different cell types suggested NPC differentiation into neurons and pre-glial cells at W5 and local neurogenesis and gliogenesis preceding migration at W8 (Fig. 4f). At W12, strong connections among neurons and glia suggested that the major events had shifted from NPC differentiation to the formation of neural circuits. In addition, TFs and cell–cell interaction analysis revealed other regulatory networks such as top TFs in each cell type and cell–cell integrations via the most significant ligand–receptor interactions (Supplementary Fig. 4). This network analysis was in line with our in situ data showing co-localization of NPCs and neural cell markers at early developmental stages (Fig. 3b,f and Extended Data Fig. 8c).

## Comparison of mouse and human spinal cord development

**Fig. 5:**
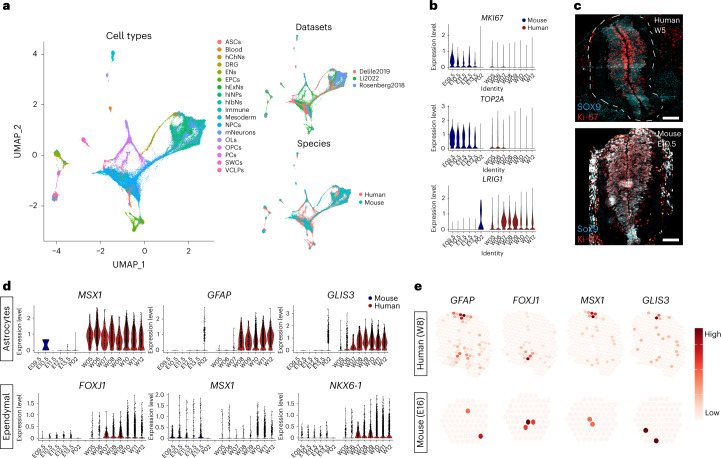
Species-specific events during neurodevelopment. **a**, UMAP illustrating integrated scRNA-seq datasets of human and mouse spinal cord development. Li2022: dataset in this study. Other two datasets: publicly available mouse development datasets. **b**, Violin plots displaying normalized gene expression of proliferation markers *MKI67* and *TOP2A* as well as stem cell quiescence regulator *LRIG1* during mouse and human spinal cord development. **c**, Representative confocal images illustrating proliferative human and mouse NPCs at early stage. Two independent experiments were performed. Scale bar, 100 μm. **d**, Violin plots displaying species differences in gene expression of gliogenesis regulators. **e**, ST plots displaying human–mouse species differences of spatial gene expression of gliogenesis regulators in the developing spinal cord.

Although most NPCs are thought to proliferate extensively before gliogenesis starts^1^, we found that more than half of the NPC clusters expressed low levels of S or G-to-M phase cell cycle genes (Extended Data Fig. 6c). To address whether low proliferation is a specific phenotype in human NPCs, we integrated our scRNA-seq datasets with two mouse spinal cord development datasets^19,20^ for comparison (Fig. 5a). In contrast to the majority of hNPCs that had low expression of proliferation markers *MKI67* and *TOP2A* from W5 to W7, mouse NPCs (mNPCs) were highly proliferative at least up to embryonic day (E) 13.5 (equivalent to human W7) (Fig. 5b). The NPC quiescence regulator *LRIG1* (ref. ^21^) also showed higher expression during embryonic and fetal stages in human compared to mouse, in which high expression took place postnatally (Fig. 5b). In agreement, IHC showed that many SOX9^+^ hNPCs were not expressing Ki-67 in the W5 human spinal cord, different from mouse E10.5 spinal cord containing mostly Sox9^+^Ki-67^+^ cells. This confirms that most hNPCs, in contrast to mNPCs, enter quiescence during early development (Fig. 5c).

Recent work revealed that ASCs and OPCs are derived as early as gestational week 8 (equivalent to W6–7 in this study) in the developing human spinal cord^6^. Our HybISS showed that glial lineage-associated genes were expressed as early as W5 (*MSX1* in ASCs, *OLIG1* and *OLIG2* in OPCs and *FOXJ1* in EPCs), equivalent to E11 in mouse development, earlier than the first appearance of mouse ASCs, OPCs and EPCs at E16.5, E12.5 and E15.5, respectively^22–25^, indicating an earlier onset of gliogenesis in human. Interestingly, *Msx1* is a key regulator of EPC differentiation during mouse spinal cord development^26^, and we found that *MSX1* is both a cell marker and a lineage-associated gene for human ASCs (Figs. 1c, 2a and 3e,f). In the human–mouse integrated scRNA-seq dataset, we found that *MSX1* was indeed highly expressed in *GFAP*-expressing ASCs in human but low in mouse ASCs (Fig. 5d). In contrast, *MSX1* and *FOXJ1* were expressed in both mouse and human EPCs, suggesting that *MSX1* has dual roles in regulating cell fate commitment of human ASC and EPC but only regulates EPCs in mice. ST analysis of human (W8) and mouse (E16) spinal cord sections at comparable timepoints validated that *MSX1* expression in humans was mainly located in the dorsal ventricular zone, correlated with the marker gene expression *GFAP* in ASCs and *FOXJ1* in EPCs (Fig. 5e). However, in mouse sections, *Msx1* was found to be expressed in the same area as *Foxj1*^+^ cells, but not in *Gfap*^+^ area, in line with a previous study^26^ showing that *Msx1* regulates mouse EPC development (Fig. 5e). In addition, we compared the most important TF activities in human and mouse (Extended Data Fig. 9a,c) and identified some regulons and their gene expression specific to humans (Extended Data Fig. 9a), such as *GLIS3* in human ASCs and NKX6-1 in human EPCs (Fig. 5d). ST confirmed that *GLIS3* was associated with *GFAP*^+^ area in human but not mouse developing spinal cord (Fig. 5e). Altogether, our data suggest that, despite the conserved mechanisms, there are fundamental differences of spatiotemporal gene expression between mouse and human spinal cord development.

## Neurodevelopment reveals tumor-specific gene expression

**Fig. 6:**
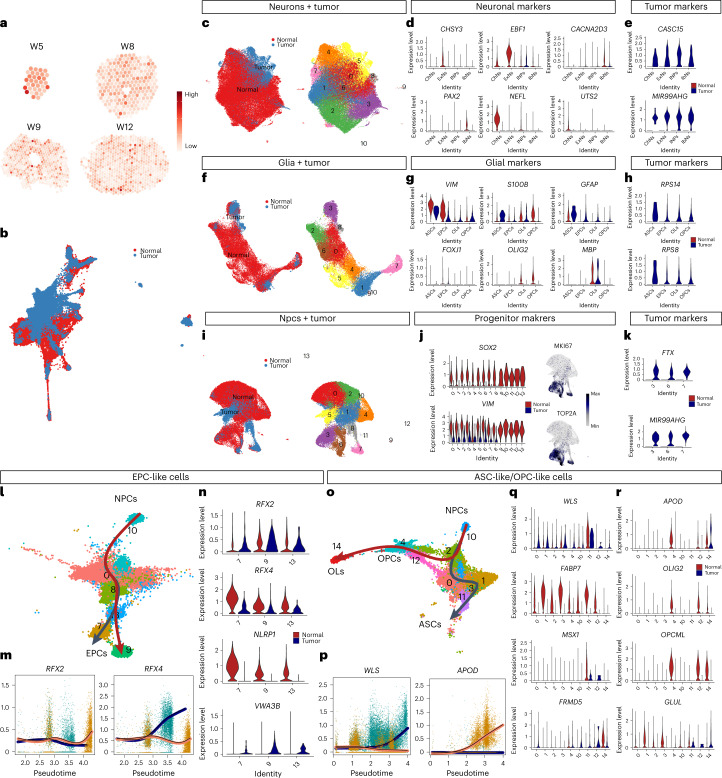
Fetal human spinal cord and relation to ependymomas. **a**, ST plots displaying spinal cord tumor gene module expression. **b**, UMAP displaying integrated normal human spinal cord and ependymomas scRNA-seq datasets. **c**–**e**, Clusters of neuronal populations shared between conditions (**c**) and the expression of normal neuronal markers (**d**) and tumor-specific markers (**e**). **f**–**h**, Clusters of glial populations shared between conditions (**f**) and the expression of normal glial markers (**g**) and tumor-specific markers (**h**). **i**–**k**, Clusters of progenitor populations shared between conditions (**i**) and the expression of normal stem cell markers (**j**) and tumor-specific markers (**k**). **l**–**n**, Trajectory analysis of EPC-like cells (**l**) and lineage-associated gene expression along pseudotime (**m**) or among branch-related clusters (**n**). **o**–**r**, Trajectory analysis of ASC-like and OPC/OL-like cells (**o**) and lineage-associated gene expression along pseudotime (**p**) or among branch-related clusters (**q**–**r**).

To demonstrate how our developmental cell atlas can be used for disease studies, we focused on ependymomas, an aggressive CNS tumor group with high recurrence rate^27,28^, especially in children^29^. Pediatric ependymoma development recapitulates neurodevelopment^27,28^, but previous scRNAseq studies lacked proper normal human neurodevelopment datasets as control^27,28^. We first used our data to gain insight into the molecular signature and differentiation of drug-resistant CSCs in pediatric ependymomas. We obtained genes related to spinal cord tumor (HP:0010302) from the Human Phenotype Ontology (HPO) database and plotted the module on ST data. We observed broad but no regionally specific gene module expression of spinal cord tumor in all ST sections (Fig. 6a), suggesting that many cell types in normal human developing spinal cord share similarities with tumors. We integrated our scRNA-seq data with human pediatric ependymomas^28^ (Fig. 6b and Supplementary Fig. 6a). Despite different overlap in neurons and glia (Fig. 6c,f), neuronal markers were predominantly expressed in the normal neurons (Fig. 6d), whereas the glial markers were similar between normal and tumor cells (Fig. 6g). The non-overlapping area probably represents biological differences between conditions. It is usually challenging to separate tumor and normal cells to identify cancer-specific biomarkers for diagnosis and treatments. Therefore, we focused on comparing the overlapping clusters between normal and tumor and identified tumor-specific genes such as *CASC15* and microRNA *MIR99AHG* in neuron-like ependymomas and *RPS14* and *RPS8* in glia-like ependymomas (Fig. 6e,h). Moreover, many CSCs overlapped with normal NPCs (Fig. 6i) and shared expression of the classical NPC markers *SOX2* and *VIM* (Fig. 6j). After identifying the putative CSC marker-associated clusters and proliferative clusters (clusters 3, 6 and 7) (Supplementary Fig. 6c and Fig. 6j), we uncovered the CSC-specific markers *FTX* and *MIR99AHG*, which were not expressed in the normal hNPCs and, thus, could be novel therapeutic targets for the ependymoma CSCs (Fig. 6i–k). We also plotted the CSC-specific genes (for example, *FTX* and *MIR99AHG)* in the ST dataset from normal spinal cord but did not find any expression in the sections, confirming that these genes are tumor specific.

Ependymoma-derived CSCs mimic neurodevelopment^28^. To further investigate the molecular differences between hNPC and CSC differentiation, our trajectory analysis showed that EPC-related TFs *RFX2* and *RFX4* were highly associated with EPC differentiation (Fig. 6l,m) in both conditions. By screening the top lineage-associated genes (Supplementary Fig. 6b), we found that *NLRP1* and *VWA3B* were specifically associated with the differentiation into normal EPCs and EPC-like CSCs, respectively (Fig. 6n). Similarly, *WLS* and *APOD* were associated with the differentiation of ASC and OPC populations (normal and tumor), respectively (Fig. 6o,p). However, normal glial cells and glia-like tumor cells have their specific lineage-associated genes, such as *FABP7* and *MSX1* in normal ASCs, *OLIG2* and *OPCML* in normal OPCs and OLs, *FRMD5* in ASC-like ependymomas and *GLUL* in OPC/OL-like ependymomas (Fig. 6q,r and Supplementary Fig. 6b). Altogether, our human spinal cord developmental atlas provides new insights of potential diagnostic or therapeutic strategies in human CNS tumors.

## An integrated spinal cord atlas across rodents and humans

**Fig. 7:**
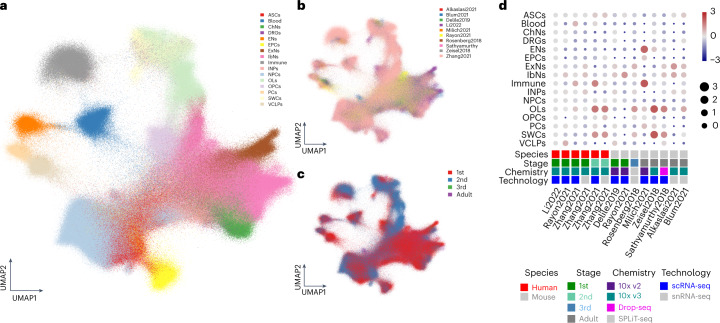
An integrated atlas of spinal cord cell types in rodents and humans. **a**–**c**, UMAP illustrating the integrated spinal cord scRNA-seq dataset with cell types (**a**) and across datasets (**b**) and developmental stages (**c**). **d**, Dot plot illustrating cell proportions across different species, developmental stages, cell capturing chemistry and technologies.

To create a spinal cord cell atlas across species, timepoints and technologies, we integrated our human scRNA-seq data with all publicly available scRNA-seq datasets of spinal cord samples as of June 2022 for 1.8 million cells (Fig. 7a–c), including human development^6,7^, mouse development^19^, mouse postnatal^20^ and adulthood^30–34^ and datasets suggested in a meta-analysis^35^. We compared our cell type annotation to the original annotation from several datasets and found high correlations (Supplementary Fig. 7). We performed label transferred from our annotated cell types to the integrated dataset (Fig. 7a) and found that our dataset (Li2022) shared high similarity with other comparable datasets of mouse and human development (Fig. 7d). Notably, the Zhang2021 dataset includes some samples from the second trimester of human spinal cord development, but not much difference in OPCs and EPCs was found, suggesting a continuation of glial cell differentiation but probably few newborn glial cell progenitors during the second trimester in the developing human spinal cord. This large integrated dataset is now also available together with the interactive map of our multi-omics data by using TissUUmap^36^ (https://hdca-sweden.scilifelab.se/tissues-overview/spinal-cord/).

## Discussion

In this study, using multi-omics and data integration to study the developing human spinal cord, we (1) created a developmental cell atlas of the human spinal cord throughout the first trimester of development; (2) revealed spatiotemporal regulation of human spinal cord neurogenesis and gliogenesis; (3) presented major differences of cell and molecular regulation between rodent and human spinal cord development; and (4) discovered unique markers and regulation of CSC differentiation in human ependymomas.

The dynamics and molecular regulation of the human spinal cord development are still understudied. Although two recent studies explored the developing human spinal cord by scRNA-seq and showed neural patterning and neurogenesis in identified clusters, they did not elucidate how NPCs are committed to multiple neural cell lineages or how the spatiotemporal gene expression is involved in neurogenesis and gliogenesis^6,7^. In this study, we acquired human prenatal spinal cords over the first trimester for scRNA-seq and spatial techniques, integrated the multi-omics datasets and validated the results, which gave new insights into the spatiotemporal gene expression of the developing human spinal cord.

NPCs are thought to proliferate vividly during fetal development^1^. However, we found that many hNPCs throughout the ventricular zone did not proliferate even at the early embryonic stage. The proliferative NPCs lose their proliferation during the first trimester in humans, much earlier than in rodents. The loss of active NPCs after fetal development limits regeneration in the mammalian adult spinal cord—for example, after spinal cord injury^24^. The loss of active NPCs during first trimester development in humans partly explains the extremely low regenerative potential in human spinal cord.

Because lineage tracing techniques cannot be applied in humans, it is unclear how neurogenesis and gliogenesis in human spinal cord are regulated in a spatiotemporal manner. With integration of multi-omics data, we highlighted some unique developmental events in the human developing spinal cord. First, we found that hNPCs were committed to glial fates as early as W5, whereas previous studies on active regulons and marker expression showed that this occurred at W8–10 (refs. ^1,24,37^). Second, whereas rodent astrocytes migrate horizontally during development to the mantle zone and the future lateral white matter^37^, we showed that human astrocytes were first restricted to the dorsal region of the spinal cord and spatiotemporally regulated by *MSX1*, a TF shown to specifically regulate ependymal cell development in rodents^26^. Third, we conclude that human EPCs exhibit a longer developmental period than expected. Mouse spinal cord EPCs are derived from mid-late fetal stage (E15.5) and are fully developed within 1 week in vivo^24^. However, whereas human EPCs are derived ventrally from W5, dorsal EPCs found at adulthood^38^ were still missing at W12, suggesting a second wave of gliogenesis during the second trimester. Future studies involving scRNA-seq and spatial techniques are needed to fully describe gliogenesis in the human spinal cord. Notably, between human and mouse development, many regulons and genes are only present in human spinal cord but not in mice, suggesting that neurodevelopment is regulated differently between species. Notably, although most studies on neurogenesis and gliogenesis have focused only on the temporal gene expression, we developed a method to demonstrate that neural patterning and positioning of neural cells are the results of the spatially biased expression in addition to temporal gene expression.

Finally, we applied our developmental atlas of the human spinal cord to investigate gene expression in childhood spinal ependymomas, a cancer type with high recurrence rate probably due to the proliferation of drug-resistant CSCs^28^. By data integration, we identified the most significant differences of gene expression between CSCs and normal stem cells. Our results give new insights into potential targets for ependymoma diagnosis and treatments.

In conclusion, we provide a comprehensive analysis of the human first trimester spinal cord during a critical phase of cellular specification and differentiation. Although we confirm that humans and rodents share many similarities during neurodevelopment, we discovered unique developmental events in the human spinal cord. Our database will not only serve as a developmental cell atlas resource but also provide important information for research on human neurodevelopmental disorders as well as regenerative strategies and cancer treatments.

## Methods

### Ethics

The prenatal specimens were retrieved from elective routine medical abortions at the Departments of Gynecology at Danderyd Hospital and Karolinska Huddinge Hospital. Patients who had decided to terminate the pregnancy were, after their decision, asked by a midwife about donation of the prenatal tissue. Patients expressing interest were given oral and written information about the research project by a midwife before the patient decided and signed the consent form. Notably, every patient was informed that they could, at any stage, change their mind, including later destruction of donated tissue already deposited in the tissue bank. All patients were at least 18 years of age, were fluent in Swedish and described themselves as ‘healthy’ and psychologically balanced as judged by the midwife. Patients terminating the pregnancy for any type of medical-psychiatric reason were not included. The clinical staff that informed the patients and performed the abortions did not in any other way participate in this research. The specimens were transported immediately from the clinic to the dissection laboratory. Spinal cord tissue was rapidly dissected in 4 °C saline (Fresenius Kabi, B306443/01) under sterile conditions within 1–2 h after the abortion. Specific information can be found in Supplementary Table 1. The entire procedure of retrieving prenatal tissue from routine clinical abortion for the use in research projects on cell mapping and characterization during prenatal organ development, specifically including the information given to the donators, was approved first by the Swedish Ethical Review Authority, followed by an independent evaluation by the National Board of Health and Welfare, as required by the Swedish regulation on the use of prenatal tissue for medical treatment and research. All procedures met the ethical stipulations of the WMA Medical Ethics Manual and the Declaration of Helsinki, and all experiments were performed in accordance with relevant guidelines and regulations. The donating patients received no compensation.

All mouse experiments were conducted in accordance with the guidelines of the Swedish Board of Agriculture (ethical permit 12570-2021) and were approved by the Karolinska Institutet Animal Care Committee.

### Human and mouse prenatal tissues

Sixteen samples of human prenatal spinal cord tissue were used in the study (13 for scRNA-seq and six for ST, HybISS and IHC), representing W5–12. In the present study, W5–8 is referred to as early stages (embryonic), and W9–12 is referred to as later first trimester stages (fetal). Post-conception age was determined by information from the clinical ultrasound, by time from last menstrual period and by identifying age-dependent anatomical landmarks with true crown-rump-length (CRL), taking into account that post-conception age and clinical age differs by 1.5–2 weeks.

Mouse fetal spinal cords were dissected quickly on ice-cold PBS after dams were sacrificed. Tissues were fresh frozen in OCT and stored for future experiments.

### Preparation of human prenatal spinal cord for multi-omics

#### scRNA-seq experiment

W5–7 spinal cord tissues were used as one piece, whereas W8–12 spinal cords were divided into three pieces (cervical, thoracic and lumbar regions) before dissociation. The dorsal root ganglia were removed by cutting the roots. Each piece of tissue was minced into smaller pieces using sterile blades and scissors. Artificial cerebrospinal (aCSF) was prepared as previously described^16^, with modification for Ca_2_Cl_2_ (1 mM) and MgCl_2_ (2 mM). The aCSF was oxygenated with 95% O_2_:5% CO_2_ for 20 min at 4 °C. The samples were then digested at 37 °C in aCSF. Papain solution (Worthington Biochemical, LK003178; 20 U ml^−1^ in CSF) and DNase I (Worthington Biochemical, LK003172; 1 mg ml^−1^) were added to the aCSF to dissociate the tissue. Incubation time was adjusted based on developmental stage, ranging from 15 min to 25 min. The spinal cords were subsequently dissociated manually with fire-polished glass pipettes. When most of the tissue was dissociated into single cells, the solution was filtered using a 30-µm cell strainer (CellTrics, Sysmex, 04-0042-2316) and collected in a 15-ml Falcon tube. The digestion solution was diluted with 7.5 ml of aCSF and centrifuged at 300*g* for 5 min at 4 °C. The pellets were resuspended in aCSF and transferred to Eppendorf tubes pre-coated with 30% BSA (Sigma-Aldrich, 9048-46-8). After cell counting, the single-cell solution was diluted to a concentration of 800–1,200 cells per microliter and kept on ice for immediate chip loading.

#### ST and ISS experiments

Human spinal cord tissues at W5, W8, W9 and W12 were embedded in Tissue-Tek (OCT) and snap frozen using an isopentane/dry ice slurry. W8–12 samples were first divided into cervical, thoracic and lumbar. To enable spatial protein and gene expression analyses, the spinal cords were cryosectioned at 16-μm thickness and alternatingly placed on Superfrost microscope glass slides (Thermo Fisher Scientific) and Visium spatial gene expression slides (10x Genomics), after which they were stored at −80 °C for no more than 14 days before being used.

#### IHC

IHC was performed as previously described^24^. In brief, tissue sections were rehydrated by 1× PBS for 5 min, and then primary antibodies diluted in blocking solution (10% normal donkey serum in PBS) were applied to the sections and incubated at room temperature overnight. Secondary antibodies were applied to sections after two times wash with 1× PBS. DAPI was applied on sections for 1 min. Sections were mounted after washing and ready for confocal imaging by Zeiss LSM 700.

### Library preparation and sequencing

#### scRNA-seq experiments

Droplet-based scRNA-seq was performed using the 10x Genomics Chromium Single Cell Kit v3. Single-cell suspensions concentrated at 800–1,200 cells per milliliter were mixed with master mix and nuclease-free water according to the Chromium manual, targeting 5,000 cells per reaction. The library preparation and sequencing were done according to the Chromium v3 standard protocol. Sequencing was performed using the Illumina NovaSeq 6000.

#### ST experiments

Spatial gene expression libraries were generated using the Visium Spatial Gene Expression Kit from 10x Genomics (https://support.10xgenomics.com/spatial-gene-expression). Sections were fixed for 30 min in methanol, stained with hematoxylin and eosin and imaged using the Metafer Slide Scanning system (MetaSystems). Optimal permeabilization time for spinal cord sections was determined to be 20 min using the 10x Genomics Visium Tissue Optimization Kit. In total, Visium Spatial Gene Expression libraries from 76 spinal cord sections were prepared by following the manufacturer’s protocol. Libraries were sequenced using Illumina platform (NovaSeq 6000 and NextSeq 2000). The number of cycles for read 1 was 28 bp and 120 bp for read 2.

#### HybISS

HybISS was performed as reported by Gyllborg et al.^5^. The protocol and materials used were as described in protocols.io (10.17504/protocols.io.xy4fpyw). Probe sequences are included in Supplementary Tables 3 and 4. For subtype/cell state markers, kits from 10x Genomics were provided along with an accompanying protocol (High Sensitivity kit). In summary, the tissue was fixed, and then the direct RNA probe mixture was added (incubated overnight at 37 °C). The section was subsequently washed, and ligation mix was added (incubated at 37 °C for 2 h). After washing, rolling circle amplification was performed at 30 °C overnight. Lastly, rounds of labeling and stripping were done for detection.

Imaging was performed with a Leica DMi8 epifluorescence microscope equipped with an LED light source (Lumencor SPECTRA X), sCMOS camera (Leica DFC9000GTC) and ×20 objective (HC PL APO, 0.80). Each field of view (FOV) was imaged with 24 *z*-stack planes with 0.5 μm spacing and 10% overlap between FOVs.

### Sequence alignment and annotation

#### scRNA-seq experiments

Single-cell sequencing data were processed using the CellRanger pipeline (version 3.0.2, 10x Genomics). Reads were mapped against the human genome (ENSEMBL genome assembly, release 93) and annotated with GENCODE gene annotations for the GRCh38-3.0.0 genome assembly (GENCODE release 32). Using the BAM files from CellRanger, molecules were mapped into spliced and unspliced transcripts using velocyto (0.17.17) into which loom files were generated for each sample.

#### ST experiments

Sequenced ST libraries were processed using the Space Ranger version 1.0.0 pipeline (10x Genomics). Reads were aligned to the human reference genome (ENSEMBL genome assembly, release 93) and annotated using GRCh38-3.0.0 to obtain expression matrixes.

### Data quality and filtering

#### scRNA-seq experiments

The single-cell count matrix was first enriched for protein-coding RNA and long intergenic non-coding RNA (lincRNA) gene types. Cells with fewer than 500 genes and genes expressed in fewer than 15 cells were excluded from the analysis. Cells with over 25% mitochondrial gene expression were also excluded.

#### ST experiments

In total, 76 tissue sections were analyzed, resulting in 20,835 spots used for data analysis. The count matrix was enriched for protein-coding and lincRNA genes. Count matrix was filtered for all hemoglobin-related genes, *MALAT1* and mitochondrial and ribosomal protein-coding genes. Spots with fewer than 500 genes and genes expressed in fewer than five spots were excluded from analysis of the three post-conception timepoints.

### Data analysis

#### Analysis for scRNA-seq and ST data

Normalization, dimensionality reduction and clustering of scRNA-seq data were performed using the Seurat package (Seurat version 4.0.4)^38^, and the top 6,000 genes with high dispersion were selected using the FindVariableGenes() function. Cell cycle activity, number of genes and mitochondrial content across the data were regressed out using the ScaleData function. Principal component analysis (PCA) was performed on the 50 most significant components as determined by the PCElbowPlot function, showing the standard deviation of the principal components. Cells in different cycling stages were identified by gene sets called ‘S.Score’ and ‘G2M.Score’ within the Seurat package. Clusters were identified using the FindClusters function by using Louvain resolution 1.2 for scRNA-seq.

Analysis, including data normalization, dimensionality reduction and clustering, of ST data was performed jointly using the Seurat and STUtility packages. Normalization was conducted using variance stabilizing transformation (SCTransform). PCA was used for selection of significant components; a total of 50 principal components were used in downstream analysis; and 30 principal components were used for ST analysis. To integrate ST sections, the Harmony (RunHarmony, version 1.0) function was used. Spots were clustered using the shared nearest neighbor algorithm implemented in the Seurat package as FindNeighbors and FindClusters (Louvain resolution 0.7).

Uniform manifold approximation and projection (UMAP) was used to create a two-dimensional (2D) embedding of cell or spot transcription profiles for visualization purposes (RunUMAP). Identification of DEGs among clusters was done using the FindAllMarkers function from the Seurat package, where genes with log fold changes (FCs) above 0.2 and *P* values below 0.01 were considered significant. For integration of scRNA-seq data and ST data, we used stereoscope^11^, which performs guided decomposition of the mixed expression data collected from each spatial capture location, using profiles learned from scRNA-seq data as a reference. In the stereoscope analysis, a batch size of 2,048 and 50,000 epochs was used for both the parameter estimation step and the proportion inference process. Cell types with fewer than 25 cells were excluded from the analysis, and we randomly selected 500 cells from cell types with more than 500 members. For cell types with more than 25 members and fewer than 500 members, all cells were included. In the analysis, 2,000 highly variable genes were used. These genes were extracted by applying the function scanpy.pp.highly_variable_genes() with n_top_genes = 2,000 from the scanpy (version 1.8.0.dev78 + gc488909a) suite, after having normalized (scanpy.pp.normalize_total(…,target_sum = 1 × 10^4^)) and log-transformed (scanpy.pp.log1p(…)) the data. Cell type decomposition of ST spots was then saved as an assay for downstream analysis.

GO characteristics of gene clusters were determined using the clusterProfiler package (version 3.8.1)^39^ for all DEGs with an average logFC value above 0 and an adjusted *P* value below 0.01. The compareCluster function was used with a pvalueCutoff = 0.05. Analysis of genes belonging to Wnt, Shh or Notch pathways as well as Human Spinal Cord development were done using the KEGG database and Phenotype Orthologs (HPO), respectively.

For previously published scRNAseq data used in this study, data sources are listed below in the ‘Data availability’ section. All these datasets were processed the same way as their publication stated.

#### Cell type annotation

After pre-processing and clustering analysis, each cluster (for both scRNA-seq and ST) was manually annotated based on previous knowledge and recent atlas resources. After annotating each cluster, clusters with the same major cell type names were merged, and DEG analysis on these major cell types was performed in an unsupervised manner. These DEG results confirmed the accuracy of annotation. In addition, all available spinal cord scRNA-seq datasets (by June 2022) were integrated, and correlation analysis for annotations was performed, which showed high correlation between our dataset annotation and previous studies.

#### Inference of branching trajectories

The R package slingshot (version 1.8.0)^15^ was used to analyze neurogenesis and gliogenesis, respectively. For neurogenesis, the NPC cluster close to INPs, all the INPs and all differentiated neurons were selected. For gliogenesis, we selected all glial cells and all NPCs that were connected to the trajectory. For each branch, clusters in the upstream and downstream were selected for pseudotime analysis. Lineage-associated genes were calculated by the R package TradeSeq (version 1.4.0)^40^.

The R package URD (version 1.1.1)^14^ was used to build differentiation trajectories during development. In the neurogenesis and gliogenesis analysis, a population of cells that were sampled from W5, clustered as NPCs_10, and with higher expression of *TOP2A* and *SOX2* was identified and used as root in the URD trajectory reconstruction. The tips of each lineage were identified based on the Louvain clusters. After 350,000 simulated random walks were performed per tip, the divergence method ‘preference’ was used to build the tree, with minimum.visits = 2, cells.per.pseudotime.bin = 25, bins.per.pseudotime.window = 8, p.thresh = 0.05 and min.cells.per.segment =10.

In the inference of hNPC development trajectory, the same population of NPCs was used as root, and the NPCs with later pseudotime estimated by scVelo and closer to neuronal and glial lineages on UMAP were identified as tips, respectively. The divergence method ‘preference’ was also used for tree building, with cells.per.pseudotime.bin = 25, bins.per.pseudotime.window = 8, p.thresh = 0.001 and other parameters default.

#### Estimation of RNA velocities

The transcriptional dynamics of splicing kinetics were modeled stochastically with scVelo (version 0.2.4)^13^ and projected onto the UMAP embedding as streamlines. To show the connectivity between different clusters, the transition probabilities of cell-to-cell transitions were estimated and projected onto the same UMAP embedding.

#### Inference of transcription factor activity

SCENIC software (version 0.11.2)^17^ was used to infer TF activities in human and mouse neural cells separately. In the human dataset, 10% of cells in each subtype were randomly sampled and combined to infer gene regulatory network with the GRNBoost2 algorithm. Then, all neural cells were used to predict candidate regulons (cisTarget) and to estimate the cellular enrichment of the predicted regulons (AUCell). The top five regulons with the highest specificity in each cell type were selected using the regulon_specificity_scores() function implemented in Python. For each regulon, its activity in all cells was fitted and binarized to determine the ‘on’ or ‘off’ state and further used to compute the ‘percent activated’ in the dot plots (Extended Data Fig. 10 and Supplementary Fig. 5).

#### Calculation of DV axis gene expression

To assess how certain feature values (for example, gene expression or cell type proportions) vary along the DV axis, we designed a method to cast the 2D data into a different and more informative one-dimensional (1D) representation relating to the aforementioned axis. More specifically, we sought to model the feature value as a function of the position along the DV axis—that is, *y*_*i*_ = *f*(*x*_*i*_), where *y*_*i*_ is the feature value of observation *i*, and *x*_*i*_ is the position of said observation on the DV axis. Below we describe in detail how we obtained the values *y*_*i*_ and *x*_*i*_ as well as the character of the non-parametric function *f*.

First, to determine each observation’s position along the DV axis, we had to define the DV axis in each sample. Thus, we manually annotated all observations (spots) as belonging to either the ventral or the dorsal region. We denoted the (mutually exclusive) sets of spots in the dorsal and ventral regions as D and V, respectively; we also let |·| represent the cardinality operator. Then, we selected a subset of observations (D′ and V′) of size min(|D|,200) and min(|V|,100), respectively, from each set and computed the ‘DV-difference vectors’ *δ*s according to:

$$\delta _s = v_k - d_s,v_k = \arg \mathop{\min}\limits_{v_k}\vert\vert d_s - v_k\vert\vert,v_k \in V^\prime ,d_s \in D^\prime$$

Whereafter we calculated the ‘average DV-difference vector’, representing the direction of the DV axis, as follows:

$$\overline {\delta _s} = \frac{1}{{\left| {D^\prime } \right|}}\mathop {\sum}\limits_s {\delta _s}$$

Finally, we let the axis vector *a* be defined as the normalized (to unit norm) average, across all observations within the sample, DV-difference vector. We then proceeded to project each observation’s spatial coordinates (in 2D space) onto the (1D) axis vector *a*, as to obtain its position along the DV axis (*p*_*s*_); for this, standard orthogonal projection is used:

$$p_s = proj_au_s = \frac{{u_s \cdot a}}{{a \cdot a}}a = \left( {u_s \cdot a} \right) \cdot a$$

Where *u*_*s*_ is an observation’s original coordinates in the 2D plane; the final equality holds true because *a* has unit norm. For each sample, we then normalized the axis projections using min–max scaling (subtraction of minimal value and division with the difference between maximal and minimal values). For computational reasons, we assign each observation *s* (based on their axis projection value) to one bin (*b*_*i*_) of *n*_*bins*_ different bins, according to:

$$p_s \ge \left( {i - 1} \right) \cdot \left( {n_{bins}} \right)^{ - 1} \wedge p_s < i \cdot \left( {n_{bins}} \right)^{ - 1} \to s \in b_i,\quad \forall i \in \left\{ {0,1, \ldots ,n_{bins}} \right\}$$

Next, for each bin *b*_*i*_, we compute the average axis value (*x*_*i*_) and average feature value (*y*_*i*_) as follows:

$$x_i = \frac{1}{{\left| {b_i} \right|}}\mathop {\sum}\limits_{s \in b_i} {p_s} ,y_i = \frac{1}{{\left| {b_i} \right|}}\mathop {\sum}\limits_{s \in b_i} {v_s}$$

Where *ν*_*s*_ is the feature value associated with observation *s*. In the last step, we aim to relate the feature values to the axis positions via a function *f*. The character of *f* is determined by loess regression (locally estimated scatterplot smoothing), implemented with geom_smooth(…, method = loess) from the R package ggplot2 and visualized as a 1D plot—generating the plots similar to those shown (for example) in Fig. 3h.

We implemented this method in R, and all code is available at GitHub (https://github.com/almaan/axis-projection) as a package that can be installed and used in a standard R environment.

#### Image processing and decoding for HybISS data

After imaging, Leica LAS X software was used to maximum intensity project each FOV to obtain a flattened 2D image. Imaging data were then analyzed with in-house custom software that handles image processing and gene calling based on the Python package Starfish. Each 2D FOV was exported and pre-processed by alignment between cycles and stitched together using the MIST algorithm^41^. Stitching was followed by retiling to create smaller non-overlapping 2,000 × 2,000 pixel images that were then used for decoding. The decoding pipeline can be found on the Moldia GitHub page (https://github.com/Moldia/iss_starfish/).

#### Probabilistic cell typing for HybISS data

Probabilistic cell maps were created using probabilistic cell typing by in situ sequencing (pciSeq). The pciSeq pipeline can be found at https://github.com/acycliq/pciSeq and is described in Qian et al.^12^. In short, pciSeq works by assigning genes to cells and then cells to cell types, and this assignment is done using a probabilistic framework based on scRNA-seq data^12^. Owing to the density of nuclei in the tissue, nuclear segmentation could not be done; instead, a compartment-based approach was employed in which each compartment was defined as a 40 × 40 pixel grid (roughly 13 × 13 µm).

### Quantification and statistical analysis

Significance of scRNA-seq and ST analysis for differential gene expression were carried out using Wilcox. Genes with *P* < 0.001 were selected as significantly differentially expressed genes. Significantly differentially expressed gene lists were ordered and filtered by smallest *P* value and largest change of logFC.

### Reporting summary

Further information on research design is available in the Nature Portfolio Reporting Summary linked to this article.

## Online content

Any methods, additional references, Nature Portfolio reporting summaries, source data, extended data, supplementary information, acknowledgements, peer review information; details of author contributions and competing interests; and statements of data and code availability are available at 10.1038/s41593-023-01312-9.

## Supplementary information

Supplementary Information Supplementary Figs. 1–7 and Supplementary Tables 1–4

Reporting Summary

Supplementary Table Table 1. Sample information of human prenatal spinal cords used in this study. Table 2. Reagents and resources used in this study. Table 3. Summary for HybISS oligo information for major cell type validation. Table 4. Summary for HybISS oligo information for subtype validation.

## Data Availability

Single-cell and spatial transcriptomics datasets produced in this manuscript are available in the Gene Expression Omnibus under accession GSE219122. The publicly available data used in this study are available at: Sathyamurthy: https://www.ncbi.nlm.nih.gov/geo/query/acc.cgi?acc=GSE103892 Zeisel: https://www.ncbi.nlm.nih.gov/sra/SRP135960 Rosenberg: https://www.ncbi.nlm.nih.gov/geo/query/acc.cgi?acc=GSE110823 Blum: https://www.ncbi.nlm.nih.gov/geo/query/acc.cgi?acc=GSE161621 Alkaslasi: https://www.ncbi.nlm.nih.gov/geo/query/acc.cgi?acc=GSE167597 Delile: https://www.ebi.ac.uk/arrayexpress/experiments/E-MTAB-7320/files Rayon: https://www.ncbi.nlm.nih.gov/geo/query/acc.cgi?acc=GSE171892 Milich: https://www.ncbi.nlm.nih.gov/geo/query/acc.cgi?acc=GSE162610 Zhang: https://www.ncbi.nlm.nih.gov/geo/query/acc.cgi?acc=GSE136719 Gojo (ependymomas): https://www.ncbi.nlm.nih.gov/geo/query/acc.cgi?acc=GSE141460

## Code availability

Codes for analysis of this paper can be found at https://github.com/czarnewski/human_developing_spinal_cord.
