# Supplementary material for: Profiling spatiotemporal gene expression of the developing human spinal cord and implications for ependymoma origin
Source: Nat Neurosci. 2023 Apr 24;26(5):891–901. doi: 10.1038/s41593-023-01312-9 (PMC10166856; doi:10.1038/s41593-023-01312-9)
Supplement: Supplementary file 1 — Supplementary Figs. 1–7 and Supplementary Tables 1–4 [file 41593_2023_1312_MOESM1_ESM.pdf]

nature neuroscience

Resource

https://doi.org/10.1038/s41593-023-01312-9

# Profiling spatiotemporal gene expression of the developing human spinal cord and implications for ependymoma origin

In the format provided by the authors and unedited

Supplementary Figure 1

HybISS panel for major cell type validation

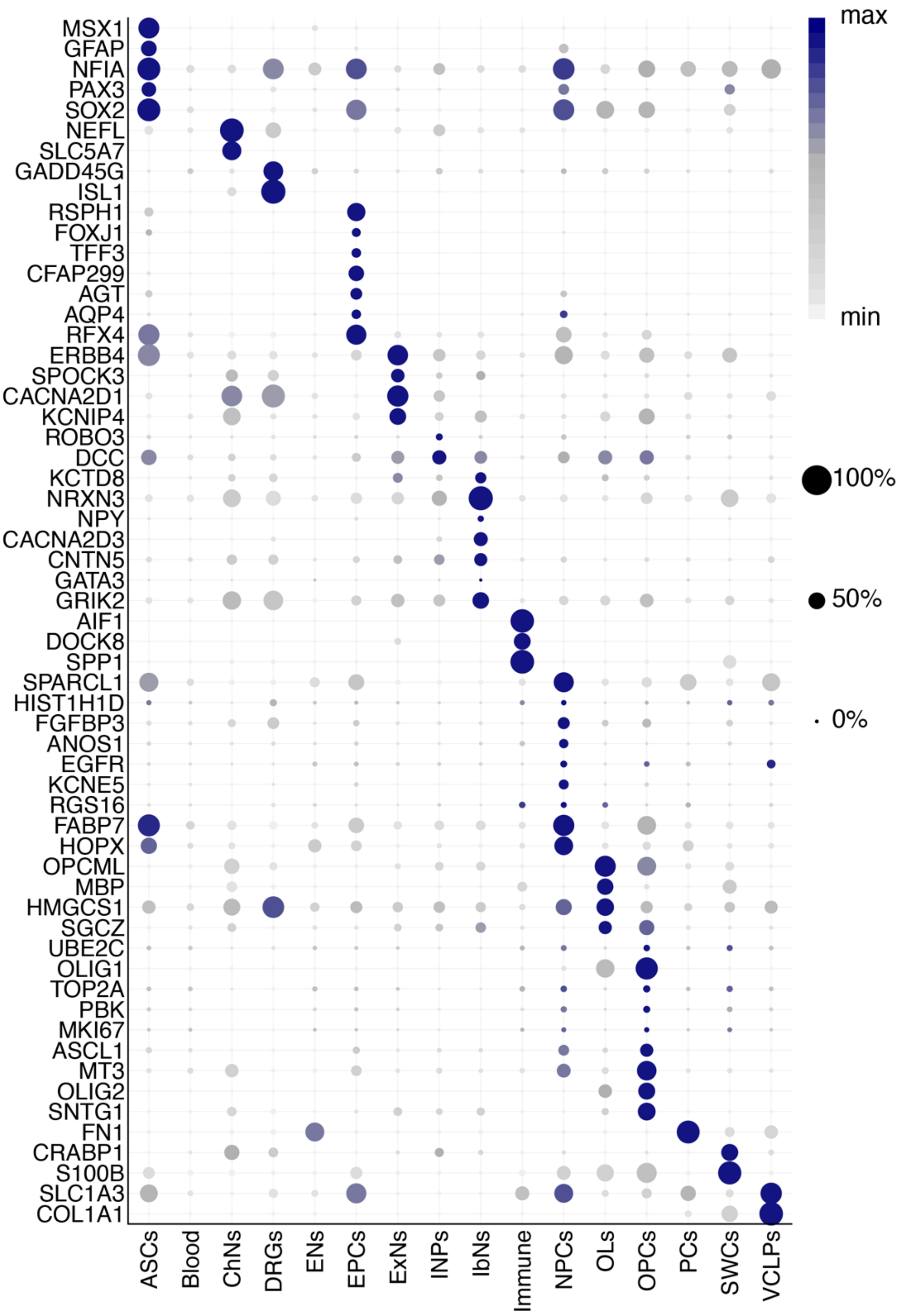


**Supplementary Figure 1. Selection of probes for HybISS based on major cell type markers.** Dot plot illustrating the correlation between major cell types from scRNA-seq and the chosen probes for validation with HybISS.

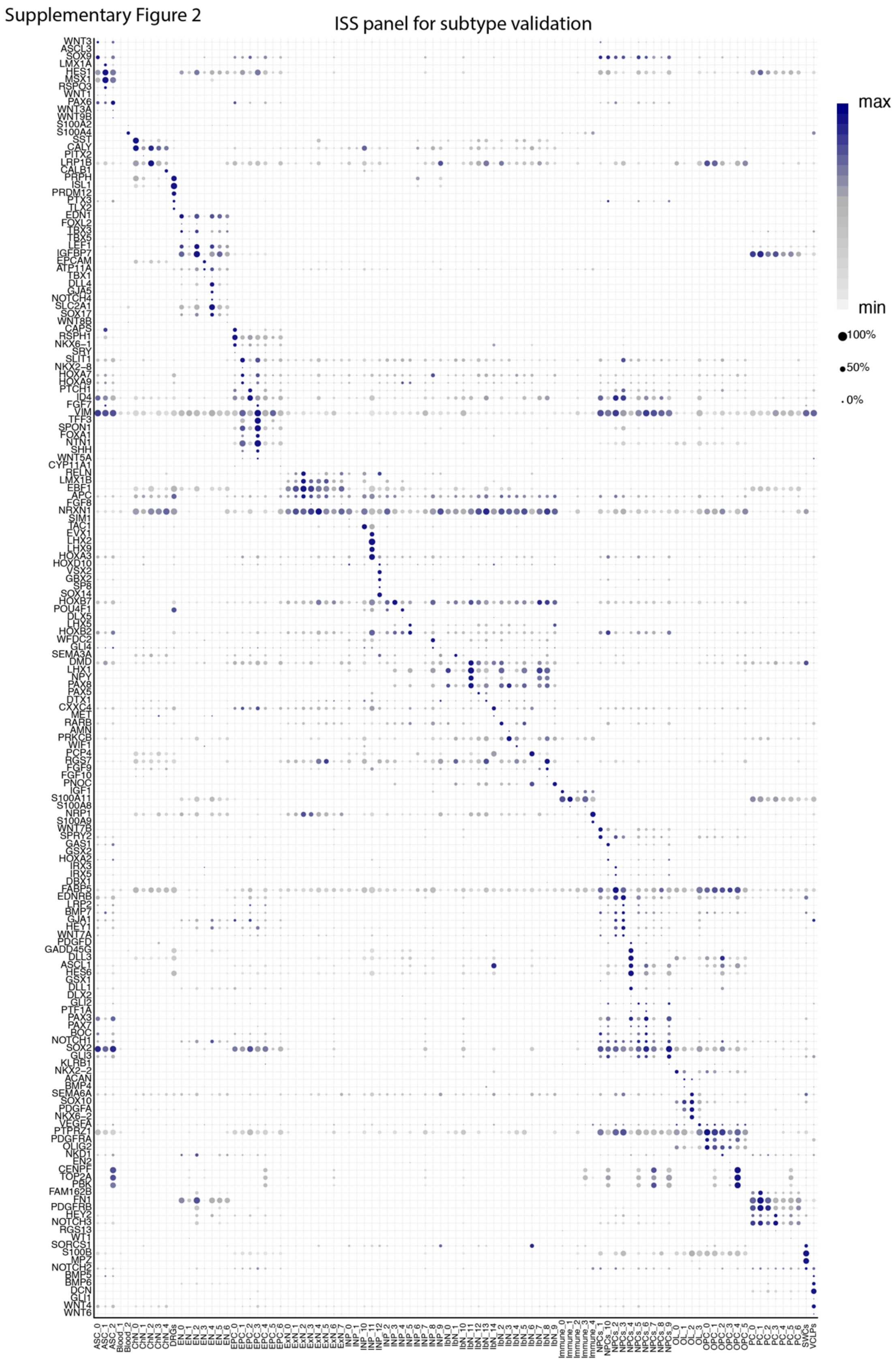


**Supplementary Figure 2. Selection of probes for HybISS based on subpopulations of each major cell type markers.** Dot plot illustrating the correlation between subtypes or cell states within each major cell type from scRNA-seq and the chosen probes for validation with HybISS.

Supplementary Figure 3

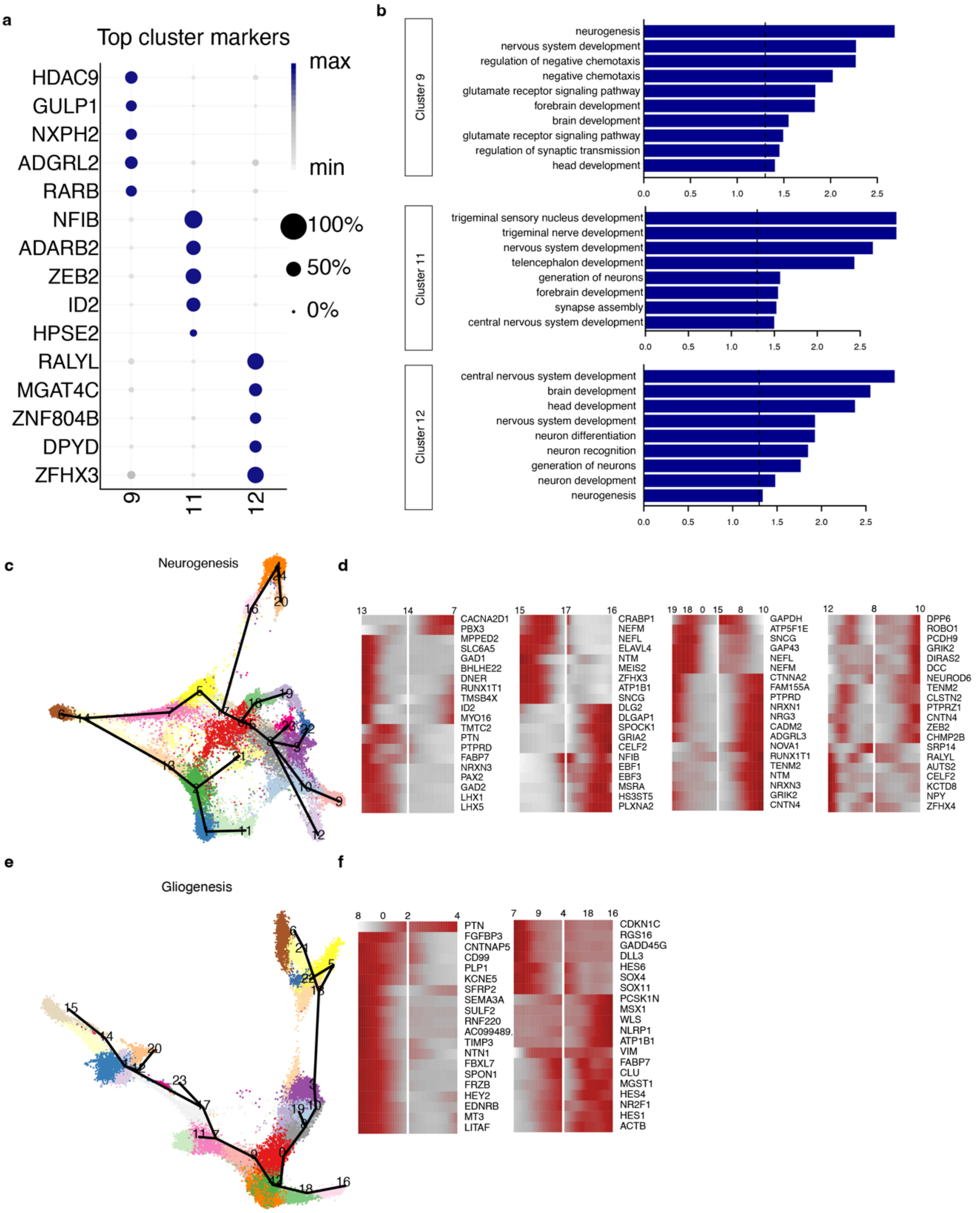


**Supplementary Figure 3. Spatiotemporal gene expression regulates neurogenesis and gliogenesis**. a) Dot plot illustrating different markers among three lineages of IbNs. b) GO terms of three terminal clusters of IbNs. c) Minimal spanning tree (MST) displaying the strongest connections between clusters related to neurogenesis. d) Heatmaps illustrating lineage differential gene expression of each branch. e) MST displaying the strongest connections between clusters related to gliogenesis. f) Heatmaps illustrating lineage differential gene expression of each branch.

Supplementary Figure 4

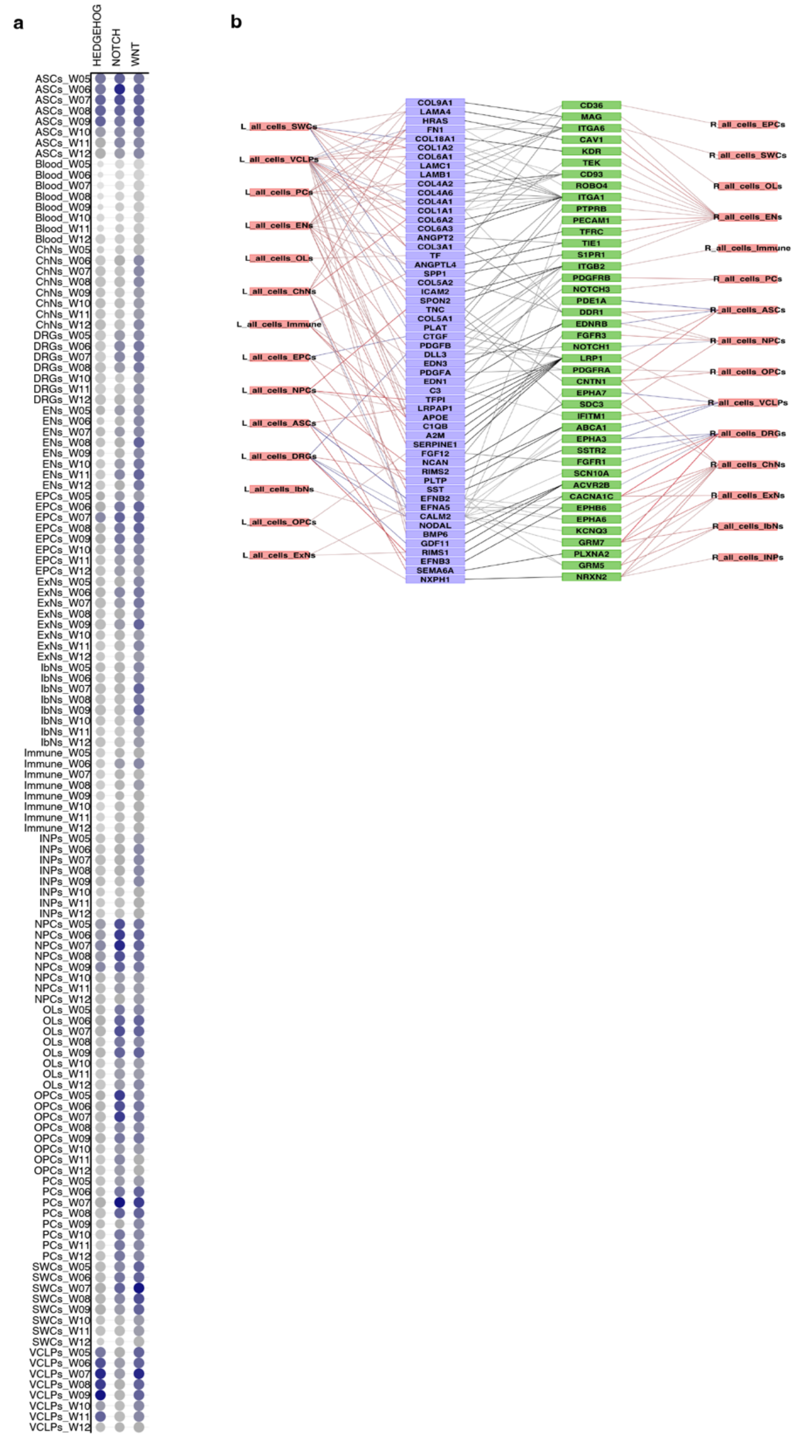


**Supplementary Figure 4. The regulatory networks of human spinal cord development.** a) Dot plot illustrating the expression of signaling pathway genes in all major cell types throughout W5-12 developmental stages. b) Interactome analysis indicate the interaction between every two cell types and the ligands and receptors contributing to the interactions. L = ligand, R = receptor. Red lines indicate increased expression of related ligands or receptors, while blue lines indicate decreased expression of related ligands and receptors during development. Thicker lines between ligands and receptors indicate higher probability of connections and cell-cell interactions.

Supplementary Figure 5

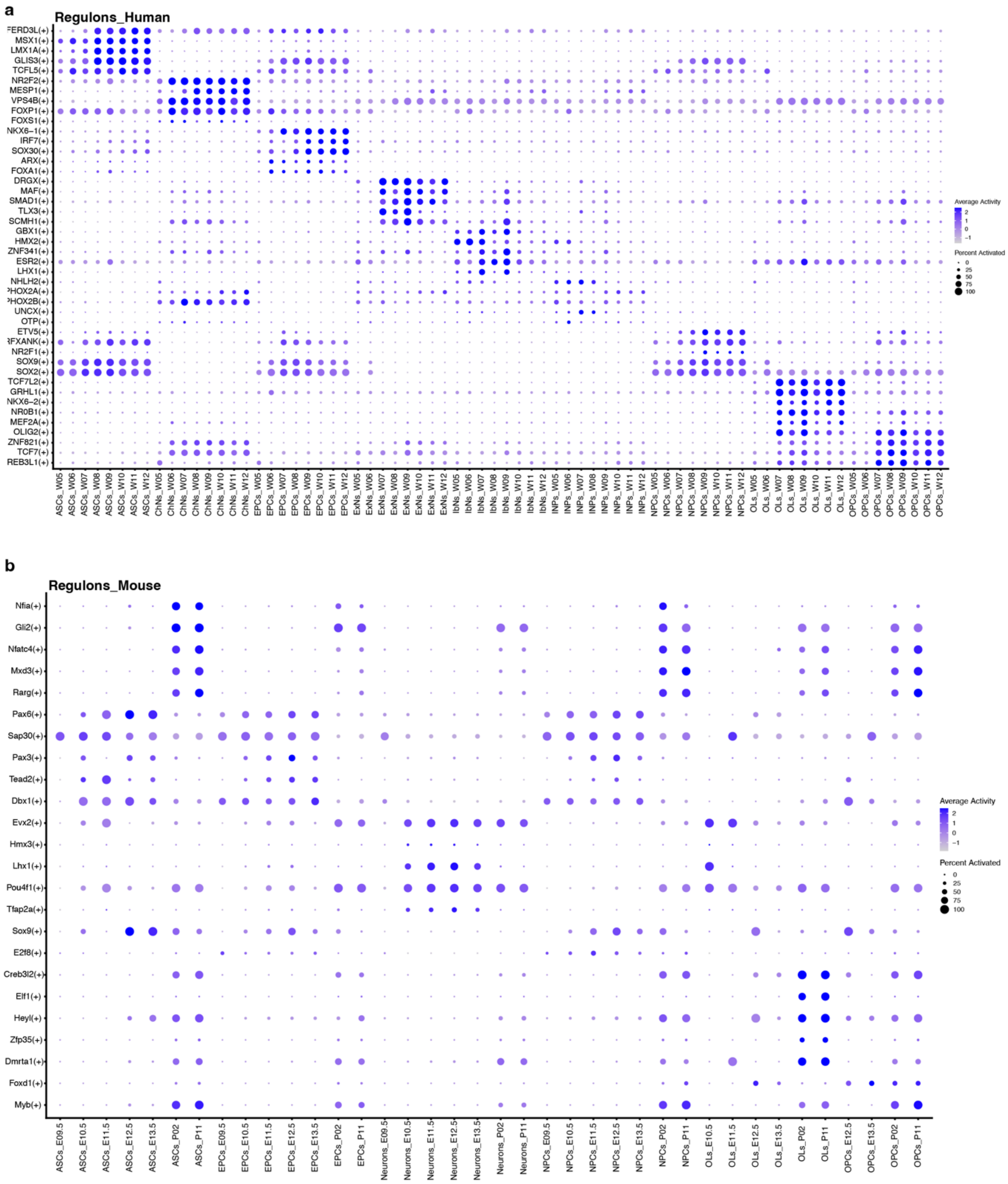


**Supplementary Figure 5. Top regulons during human and mouse spinal cord development.** a) Dot plot illustrating the most significant regulons in the developing human spinal cord during W5-12 across major cell types and age. b) Dot plot illustrating the most significant regulons in the developing mouse spinal cord from E9.5-P11 across major cell types and age.

Supplementary Figure 6

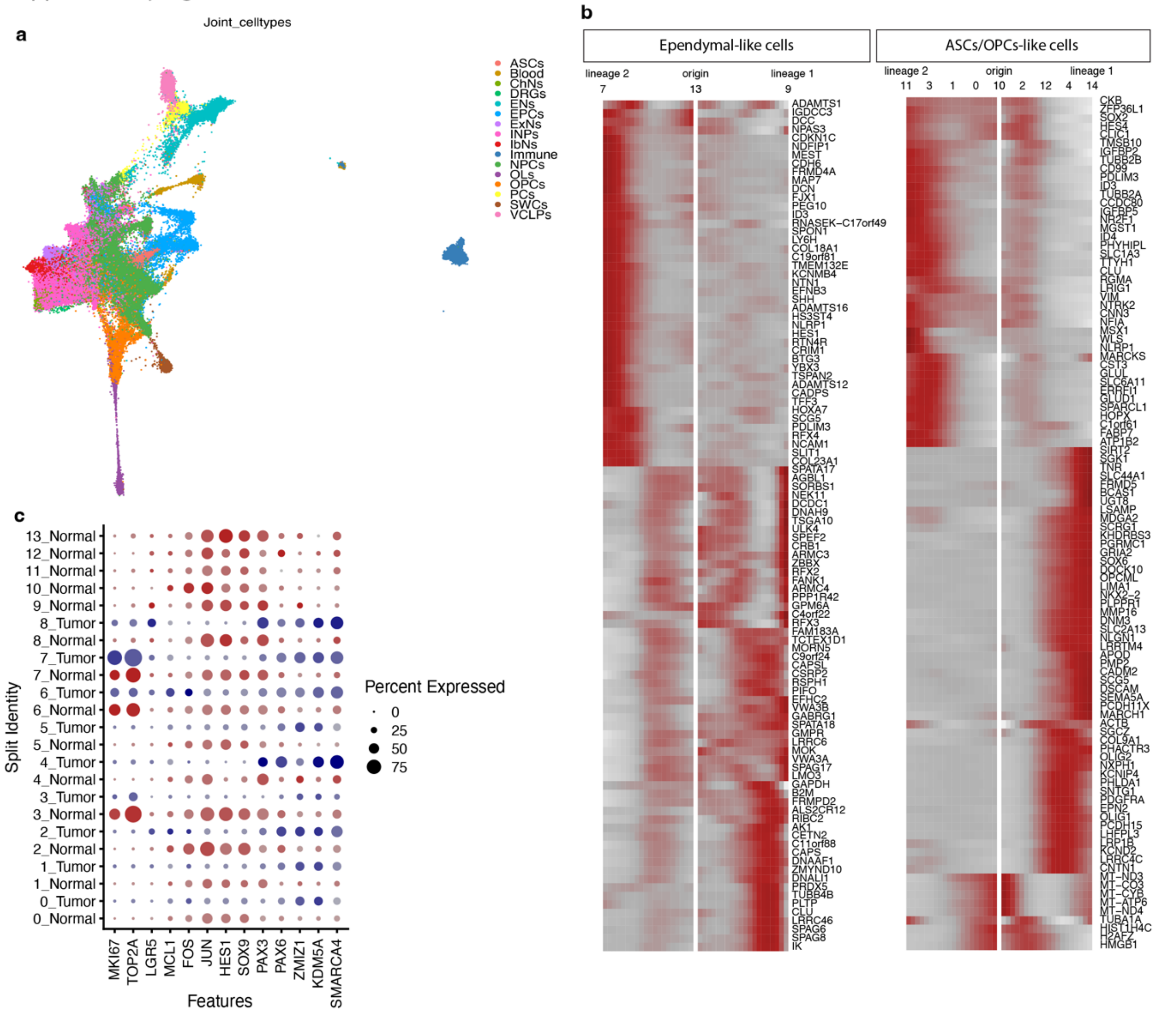


**Supplementary Figure 6. Fetal human spinal cord and relation to ependymomas.** a) UMAP displaying all major cell types revealed by the integrated scRNA-seq dataset of human developing spinal cord and human ependymomas. b) Heatmaps revealing the most significantly differential expressed genes in two lineages of EPCs-like cells and ASCs and OPCs/OLs-like cells during trajectory analysis. c) Dot plot illustrating the expression of putative cancer stem cell marker genes across all subtypes of human NPCs and human CSCs and is selectively enriched in cluster 3, 6, and 7.

Supplementary Figure 7

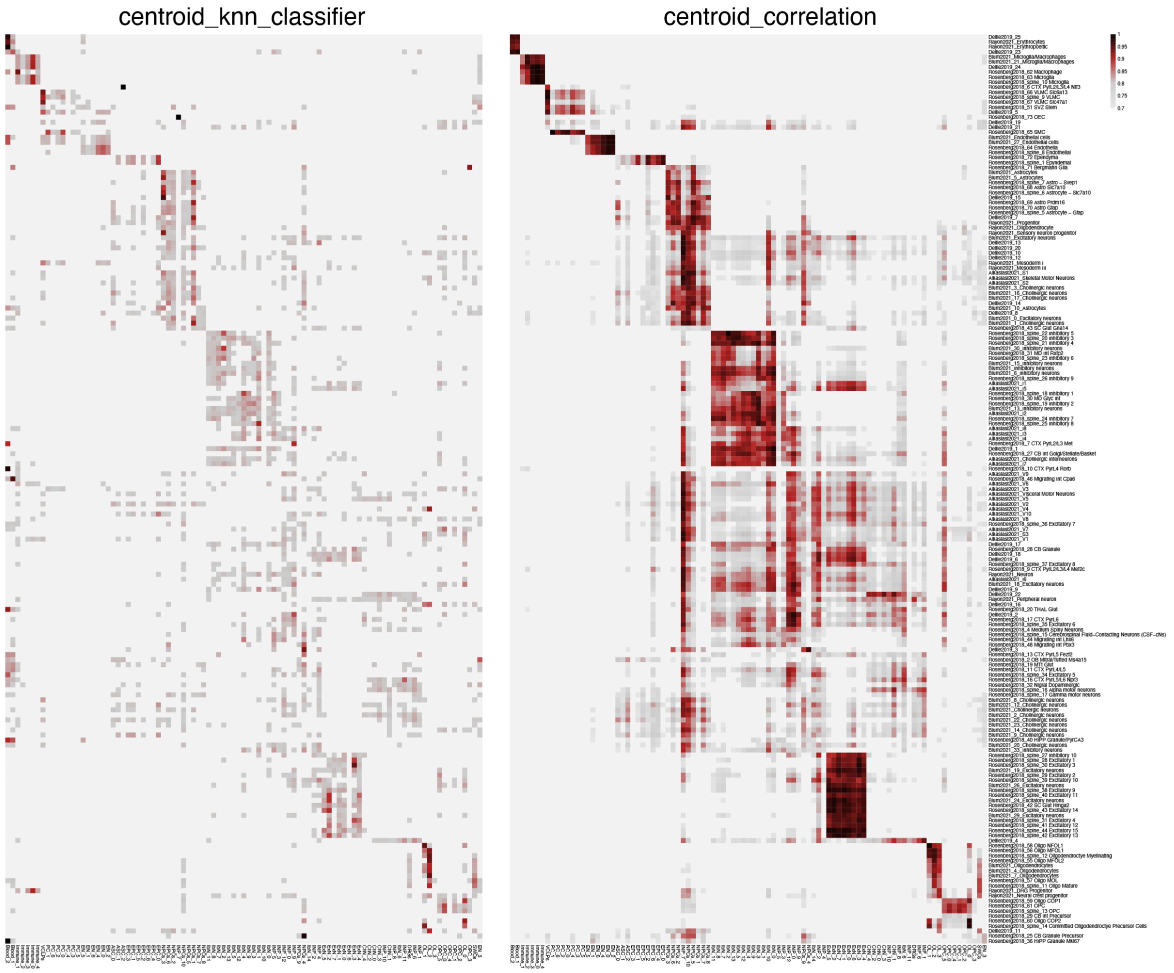


**Supplementary Figure 7. Correlation with annotations from previous spinal cord datasets.** Heatmaps illustrating good correlation between the annotation of scRNA-seq data from this study with previous scRNA-seq data by two different correlation calculations.
