# Supplementary material for: Profiling spatiotemporal gene expression of the developing human spinal cord and implications for ependymoma origin
Source: Nat Neurosci. 2023 Apr 24;26(5):891–901. doi: 10.1038/s41593-023-01312-9 (PMC10166856; doi:10.1038/s41593-023-01312-9)
Supplement: Supplementary file 2 — Reporting Summary [file 41593_2023_1312_MOESM2_ESM.pdf]

nature portfolio

Corresponding author(s): Xiaofei Li

Last updated by author(s): 16/02/23

Reporting Summary

Nature Portfolio wishes to improve the reproducibility of the work that we publish. This form provides structure for consistency and transparency in reporting. For further information on Nature Portfolio policies, see our Editorial Policies and the Editorial Policy Checklist.

Statistics

For all statistical analyses, confirm that the following items are present in the figure legend, table legend, main text, or Methods section.

n/a	Confirmed	
☐	☒	The exact sample size (n) for each experimental group/condition, given as a discrete number and unit of measurement
☐	☒	A statement on whether measurements were taken from distinct samples or whether the same sample was measured repeatedly
☐	☒	The statistical test(s) used AND whether they are one- or two-sided<br>*Only common tests should be described solely by name; describe more complex techniques in the Methods section.*
☒	☐	A description of all covariates tested
☐	☒	A description of any assumptions or corrections, such as tests of normality and adjustment for multiple comparisons
☐	☒	A full description of the statistical parameters including central tendency (e.g. means) or other basic estimates (e.g. regression coefficient) AND variation (e.g. standard deviation) or associated estimates of uncertainty (e.g. confidence intervals)
☒	☐	For null hypothesis testing, the test statistic (e.g. F, t, r) with confidence intervals, effect sizes, degrees of freedom and P value noted<br>*Give P values as exact values whenever suitable.*
☒	☐	For Bayesian analysis, information on the choice of priors and Markov chain Monte Carlo settings
☐	☒	For hierarchical and complex designs, identification of the appropriate level for tests and full reporting of outcomes
☒	☐	Estimates of effect sizes (e.g. Cohen's d, Pearson's r), indicating how they were calculated

Our web collection on statistics for biologists contains articles on many of the points above.

Software and code

Policy information about availability of computer code

Data collection	No software was used for data collection, other than as part of Illumina and 10x Genomics instruments
Data analysis	All custom source code is available at https://github.com/czarnewski/human_developing_spinal_cord. R 4.1.2 is used for analysis. R package: ggplot2_3.3.5, niceRplots_0.1.0, harmony_1.0, Rcpp_1.0.7, scales_1.1.1, rafalib_1.0.0, biomaRt_2.46.3, Seurat_4.0.4, STutility, Space Ranger v1.0.0. See Methods and the link (https://github.com/czarnewski/human_developing_spinal_cord) for details on how each software is used.

For manuscripts utilizing custom algorithms or software that are central to the research but not yet described in published literature, software must be made available to editors and reviewers. We strongly encourage code deposition in a community repository (e.g. GitHub). See the Nature Portfolio guidelines for submitting code & software for further information.

Data

Policy information about availability of data

All manuscripts must include a data availability statement. This statement should provide the following information, where applicable:

- Accession codes, unique identifiers, or web links for publicly available datasets
- A description of any restrictions on data availability
- For clinical datasets or third party data, please ensure that the statement adheres to our policy

The Single cell and Spatial Transcriptomics dataset produced in this manuscript are available on GEO under accession GSE219122. All datasets of this paper has been summarized at: https://github.com/czarnewski/human_developing_spinal_cord

The publicly available data utilized in this study are available at: Sathyamurthy: https://www.ncbi.nlm.nih.gov/geo/query/acc.cgi?acc=GSE103892
Zeisel: https://www.ncbi.nlm.nih.gov/sra/SRP135960
Rosenberg: https://www.ncbi.nlm.nih.gov/geo/query/acc.cgi?acc=GSE110823
Blum: https://www.ncbi.nlm.nih.gov/geo/query/acc.cgi?acc=GSE161621
Alkaslasi: https://www.ncbi.nlm.nih.gov/geo/query/acc.cgi?acc=GSE167597
Delile: https://www.ebi.ac.uk/arrayexpress/experiments/E-MTAB-7320/files
Rayon: https://www.ncbi.nlm.nih.gov/geo/query/acc.cgi?acc=GSE171892
Milich: https://www.ncbi.nlm.nih.gov/geo/query/acc.cgi?acc=GSE162610
Zhang: https://www.ncbi.nlm.nih.gov/geo/query/acc.cgi?acc=GSE136719
Gojo (ependymomas): https://www.ncbi.nlm.nih.gov/geo/query/acc.cgi?acc=GSE141460

Human research participants

Policy information about studies involving human research participants and Sex and Gender in Research.

Reporting on sex and gender	The samples were collected in a mixed gender manner. Details can be seen in the manuscript and in the available data (public link included in the manuscript) by plotting sex chromosome gene expression.
Population characteristics	All embryo and fetal tissued were between 5-12 postconceptional weeks. Postconceptional age was measured by CRL and anatomical landmarks, as well as used gestational age was measured in weeks from the first day of the woman's last menstrual cycle to the sample collecting date and ultrasound.
Recruitment	The prenatal specimens were retrieved from elective medical abortions at the Departments of Gynecology at Danderyd Hospital and Karolinska Huddinge Hospital. Patients who had decided to terminate the pregnancy were after their decision asked by a midwife about donation of the prenatal tissue. Patients expressing interest were given oral and written information about the research project by a midwife before the patient made a decision and signed the consent form. Importantly, every patient was informed that they could at any stage change their mind, including the later destruction of donated tissue already deposited in the tissue bank. All patients were at least 18 years of age and fluent in Swedish. The clinical staff that informed the patients and performed the abortions did not in any other way participate in this research. Donors that fulfill the requirements above were therefore recruited in an unbiased manner.
Ethics oversight	The use of prenatal tissue for this study was approved by the Swedish Ethical Review Authority and the National Board of Health and Welfare. All procedures met the ethical stipulations of the WMA Medical Ethics Manual and the Declaration of Helsinki, and all experiments were performed in accordance with relevant guidelines and regulations.

Note that full information on the approval of the study protocol must also be provided in the manuscript.

Field-specific reporting

Please select the one below that is the best fit for your research. If you are not sure, read the appropriate sections before making your selection.

☒ Life sciences ☐ Behavioural & social sciences ☐ Ecological, evolutionary & environmental sciences

For a reference copy of the document with all sections, see nature.com/documents/nr-reporting-summary-flat.pdf

Life sciences study design

All studies must disclose on these points even when the disclosure is negative.

Sample size	The sample size of scRNA-seq, ST and HybISS were determined by availability of human tissues and previous experience (partly from our other papers regarding human first trimester development e.g. Sountoulidis et al., Nat Cell Biology 2023; Braun et al., BioRxiv 2022; Asp et al., Cell 2019). We collected 16 spinal cords from embryonic stages for scRNA-seq with mostly 2-4 samples at the same developmental stage as replications . Final dataset scale was determined according to the quality control criteria as described in the methods.
Data exclusions	Cells detected with than 25% mitochondria genes were removed as low quality cells. Cells with the number of UMI, genes and counts in the highest and lowest 0.5% were removed.
Replication	In scRNA-seq, W5, 10 and 11 have 2-4 fetuses as representative ages for biological replicates while in ST and HybISS, W5 and W8 had 2 cases each to compared gene expression. Each sample had loaded 2-4 wells in the 10x Genomics chips for scRNA-seq as tehnical replicate. Consecutive tissue sections from the same spinal cord tissue were considered technical replicates in the ST and HybISS experiments. However, it is important to notice that consecutive sections are highly similar but not identical.
Randomization	The samples were allocated into each experimental groups based on the postconceptional stage. See methods 'Human developmental tissue'.
Blinding	The investigators were blinded to group allocation during data collection and analysis.

Reporting for specific materials, systems and methods

We require information from authors about some types of materials, experimental systems and methods used in many studies. Here, indicate whether each material, system or method listed is relevant to your study. If you are not sure if a list item applies to your research, read the appropriate section before selecting a response.

Materials & experimental systems

n/a	Involved in the study
☐	☒ Antibodies
☒	☐ Eukaryotic cell lines
☒	☐ Palaeontology and archaeology
☐	☒ Animals and other organisms
☒	☐ Clinical data
☒	☐ Dual use research of concern

Methods

n/a	Involved in the study
☒	☐ ChIP-seq
☒	☐ Flow cytometry
☒	☐ MRI-based neuroimaging

Antibodies

Antibodies used

Mouse monoclonal anti-NKX2.2, 74.5A5, DSHB, 1:50
Mouse monoclonal anti-SHH, 5E1, DSHB, 1:20
Mouse monoclonal anti-active-β-CATENIN, clone 8E7, cat, no. 05-665, Sigma-Aldrich, 1:1000
Mouse monoclonal anti-ISL1, 40.3A4, DSHB, 1:100
Rabbit anti-hPDGFRα, cat. no. 5241, Cell Signaling Technology, 1:300
Rabbit anti-GFAP, cat. no. Z0334, DAKO, 1:1000
Rabbit anti-OLIG2, cat. no. AB9610, Millipore, 1:1000
Goat anti-OLIG2, cat. no. AF2418, R&D systems, 1:500
Rabbit monoclonal anti-HES1, clone D6P2U, cat. no. 11988, Cell Signaling Technology, 1:2000
Rat monoclonal anti-Ki67, clone solA15, cat. no. 14-5698-82, Invitrogen, 1:1000
Goat anti-GLI3, cat. no. AF3690, R&D systems, 1:300
Rabbit anti-GLI1, cat. no. 2553, Cell Signaling Technology, 1:500
Goat anti-MSX1, cat. no. AF5045, R&D systems, 1:300
Rabbit anti-SOX9, cat. no. AB5535, Millipore, 1:1000
Rabbit RFX4, cat. no., BS-11943R, Thermo Fisher Scientific, 1:500
Goat anti-FOXJ1, cat. no. AF3619, R&D systems, 1:300
Rabbit anti-EBF-1, cat. no. AB10523, Millipore, 1:1000
Rabbit anti-PAX2, cat. no.901001, BioLegend, 1:1000
Alexa Fluor 488 donkey anti-rabbit IgG, cat. no. A21206, Invitrogen, 1:500
Alexa Fluor 555 donkey anti-rat IgG, cat. no. A48270, Invitrogen, 1:500
Alexa Fluor 488 donkey anti-mouse IgG, cat. no. A21202, Invitrogen, 1:500
Alexa Fluor 555 donkey anti-mouse IgG, cat. no. A31570, Invitrogen, 1:500
Alexa Fluor 488 goat anti-mouse IgG2b, cat. no. A21141, Invitrogen, 1:5
Alexa Fluor 555 donkey anti-goat IgG, cat. no. A21432, Invitrogen, 1:500
Alexa Fluor 555 goat anti-mouse IgG1, cat. no. A211127, Invitrogen, 1:500

Validation

Mouse monoclonal anti-NKX2.2, 74.5A5, DSHB
https://dshb.biology.uiowa.edu/74-5A5
Mouse monoclonal anti-SHH, 5E1, DSHB
https://dshb.biology.uiowa.edu/5E1
Mouse monoclonal anti-active-β-CATENIN, clone 8E7, cat, no. 05-665, Sigma-Aldrich
https://www.merckmillipore.com/SE/en/product/Anti-Active-Catenin-Anti-ABC-Antibody-clone-8E7,MM_NF-05-665?ReferrerURL=https%3A%2F%2Fwww.google.com%2F
Mouse monoclonal anti-ISL1, 40.3A4, DSHB
https://dshb.biology.uiowa.edu/40-3A4
Rabbit anti-hPDGFRα, cat. no. 5241, Cell Signaling Technology
https://www.cellsignal.com/products/primary-antibodies/pdgf-receptor-a-d13c6-xp-rabbit-mab/5241
Rabbit anti-GFAP, cat. no. Z0334, DAKO
https://www.labome.com/product/Dako/Z0334.html
Rabbit anti-OLIG2, cat. no. AB9610, Millipore
https://www.merckmillipore.com/SE/en/product/Anti-Olig-2-Antibody,MM_NF-AB9610?ReferrerURL=https%3A%2F%2Fwww.google.com%2F
Goat anti-OLIG2, cat. no. AF2418, R&D systems
https://www.rndsystems.com/products/human-mouse-rat-olig2-antibody_af2418?gclid=Cj0KCQiAxbefBhDfARIsAL4XLRoRcbH8Pd_Jib77xTQhB8zgOYfucPmw6Hq9Udveu443V4ncG2qrHCkaAtNsEALw_wcB&gclsrc=aw.ds
Rabbit monoclonal anti-HES1, clone D6P2U, cat. no. 11988, Cell Signaling Technology
https://www.cellsignal.com/products/primary-antibodies/hes1-d6p2u-rabbit-mab/11988?utm_source=google&utm_medium=cpc&utm_campaign=can&utm_content=ctla-4%20-%20mon%20-%20dynamic%20-%20cst_row%20-%

20emea&utm_term=&utm_tactic=ppc&utm_region=hq&utm_conv=tdr&utm_stage=ase&utm_seg=ind&utm_prog=scs&gclid=Cj0KCQiAxbefBhDfARIsAL4XLRrQi-LZNzP8gKE-hHQP4Xt5a6C9B2jWwIY7yo7TL-6ld7wYO7yqcjIaAgAVEALw_wcB&gclsrc=aw.ds
Rat monoclonal anti-Ki67, clone SolA15, cat. no. 14-5698-82, Invitrogen
https://www.thermofisher.com/antibody/product/Ki-67-Antibody-clone-SolA15-Monoclonal/14-5698-82
Goat anti-GLI3, cat. no. AF3690, R&D systems
https://www.rndsystems.com/products/human-mouse-gli-3-antibody_af3690?gclid=Cj0KCQiAxbefBhDfARIsAL4XLRq0FcNl7x_0DV5pq9eexbUe52HtD6Yalc5SRCylsWv0eyggOHLCqy8aAj1kEALw_wcB&gclsrc=aw.ds
Rabbit anti-GLI1, cat. no. 2553, Cell Signaling Technology
https://www.cellsignal.com/products/primary-antibodies/gli1-antibody/2553
Goat anti-MSX1, cat. no. AF5045, R&D systems
https://www.rndsystems.com/products/human-mouse-msx1-antibody_af5045

Rabbit anti-SOX9, cat. no. AB5535, Millipore
https://www.merckmillipore.com/SE/en/product/Anti-Sox9-Antibody,MM_NF-AB5535?ReferrerURL=https%3A%2F%2Fwww.google.com%2F
Rabbit RFX4, cat. no., BS-11943R, Thermo Fisher Scientific
https://www.thermofisher.com/antibody/product/RFX4-Antibody-Polyclonal/BS-11943R
Goat anti-FOXJ1, cat. no. AF3619, R&D systems
https://www.rndsystems.com/products/human-foxj1-antibody_af3619
Rabbit anti-EBF-1, cat. no. AB10523, Millipore
https://www.merckmillipore.com/SE/en/product/Anti-EBF-1-Antibody,MM_NF-AB10523?ReferrerURL=https%3A%2F%2Fwww.google.com%2F
Rabbit anti-PAX2, cat. no.901001, BioLegend
https://www.biolegend.com/de-at/products/purified-anti-pax-2-antibody-11510
Alexa Fluor 488 donkey anti-rabbit IgG, cat. no. A21206, Invitrogen
https://www.thermofisher.com/antibody/product/A-21206.html?gclid=Cj0KCQiAxbefBhDfARIsAL4XLRrtTvAB2_ld3xZgeWy5sRCFsKZmd7fgULCL_85XgbKuBeuSC-v37UsaAtrPEALw_wcB&ef_id=Cj0KCQiAxbefBhDfARIsAL4XLRrtTvAB2_ld3xZgeWy5sRCFsKZmd7fgULCL_85XgbKuBeuSC-v37UsaAtrPEALw_wcB:G:s&s_kwcid=AL!3652!3!516608152221!!!g!!!12825517856!122158235235&cid=bid_pca_aus_r01_co_cp1359_pjt0000_bid00000_0se_gaw_dy_pur_con
Alexa Fluor 555 donkey anti-rat IgG, cat. no. A48270, Invitrogen
https://www.thermofisher.com/antibody/product/A48270.html?gclid=Cj0KCQiAxbefBhDfARIsAL4XLRqEClYN0gj59cjo4LC9J-DDcPcft3hbmqTECk1hk9uK0r134EP447EaAhZfEALw_wcB&ef_id=Cj0KCQiAxbefBhDfARIsAL4XLRqEClYN0gj59cjo4LC9J-DDcPcft3hbmqTECk1hk9uK0r134EP447EaAhZfEALw_wcB:G:s&s_kwcid=AL!3652!3!516608152206!!!g!!!12825517856!122158235275&cid=bid_pca_aus_r01_co_cp1359_pjt0000_bid00000_0se_gaw_dy_pur_con
Alexa Fluor 488 donkey anti-mouse IgG, cat. no. A21202, Invitrogen
https://www.thermofisher.com/antibody/product/Donkey-anti-Mouse-IgG-H-L-Highly-Cross-Adsorbed-Secondary-Antibody-Polyclonal/A-21202?gclid=Cj0KCQiAxbefBhDfARIsAL4XLRoHb-jL1tys7npUTBRMWuQ3fS9J-B_6EgAvx1IN9WLBlQ-D7EjbvOIaAse_EALw_wcB&ef_id=Cj0KCQiAxbefBhDfARIsAL4XLRoHb-jL1tys7npUTBRMWuQ3fS9J-B_6EgAvx1IN9WLBlQ-D7EjbvOIaAse_EALw_wcB:G:s&s_kwcid=AL!3652!3!516608152458!!!g!!!12825517856!122158234995&cid=bid_pca_aus_r01_co_cp1359_pjt0000_bid00000_0se_gaw_dy_pur_con
Alexa Fluor 555 donkey anti-mouse IgG, cat. no. A31570, Invitrogen
https://www.thermofisher.com/antibody/product/A-31570.html?gclid=Cj0KCQiAxbefBhDfARIsAL4XLRoBUB1gBlY7iezwE3HVmDICC5ZNZDKmlGS8EDFWxLqvqGY9dgNrIdcaApx8EALw_wcB&ef_id=Cj0KCQiAxbefBhDfARIsAL4XLRoBUB1gBlY7iezwE3HVmDICC5ZNZDKmlGS8EDFWxLqvqGY9dgNrIdcaApx8EALw_wcB:G:s&s_kwcid=AL!3652!3!516608152455!!!g!!!12825517856!122158234995&cid=bid_pca_aus_r01_co_cp1359_pjt0000_bid00000_0se_gaw_dy_pur_con
Alexa Fluor 488 goat anti-mouse IgG2b, cat. no. A21141, Invitrogen
https://www.thermofisher.com/antibody/product/A-21141.html?gclid=Cj0KCQiAxbefBhDfARIsAL4XLRrw5rB8zR7CDwV1hOjo4cRVq4eE-9rCcwUdXDQs0PkxxT5a3JS2NZIaAg7bEALw_wcB&ef_id=Cj0KCQiAxbefBhDfARIsAL4XLRrw5rB8zR7CDwV1hOjo4cRVq4eE-9rCcwUdXDQs0PkxxT5a3JS2NZIaAg7bEALw_wcB:G:s&s_kwcid=AL!3652!3!516608152455!!!g!!!12825517856!122158234995&cid=bid_pca_aus_r01_co_cp1359_pjt0000_bid00000_0se_gaw_dy_pur_con
Alexa Fluor 555 donkey anti-goat IgG, cat. no. A21432, Invitrogen
https://www.thermofisher.com/antibody/product/A-21432.html?gclid=Cj0KCQiAxbefBhDfARIsAL4XLRoQ7DZxhaXw0v_s3TSGZADvIbNierzAGJoAblsPMnjnlcH3ylf_I-IaAmC6EALw_wcB&ef_id=Cj0KCQiAxbefBhDfARIsAL4XLRoQ7DZxhaXw0v_s3TSGZADvIbNierzAGJoAblsPMnjnlcH3ylf_I-IaAmC6EALw_wcB:G:s&s_kwcid=AL!3652!3!516608152257!!!g!!!12825517856!122158234515&cid=bid_pca_aus_r01_co_cp1359_pjt0000_bid00000_0se_gaw_dy_pur_con
Alexa Fluor 555 goat anti-mouse IgG1, cat. no. A-21127, Invitrogen
https://www.thermofisher.com/antibody/product/Goat-anti-Mouse-IgG1-Cross-Adsorbed-Secondary-Antibody-Polyclonal/A-21127

Animals and other research organisms

Policy information about studies involving animals; ARRIVE guidelines recommended for reporting animal research, and Sex and Gender in Research

Laboratory animals	c57 animals were used in the manuscript
Wild animals	None
Reporting on sex	randomized embryos were used
Field-collected samples	N.A.
Ethics oversight	The use of prenatal tissue for this study was approved by the Swedish Ethical Review Authority and the National Board of Health and Welfare. All procedures met the ethical stipulations of the WMA Medical Ethics Manual and the Declaration of Helsinki, and all experiments were performed in accordance with relevant guidelines and regulations.

Note that full information on the approval of the study protocol must also be provided in the manuscript.
